# Nitric Oxide Signaling and Sensing in Age-Related Diseases

**DOI:** 10.3390/antiox13101213

**Published:** 2024-10-09

**Authors:** Olga Mazuryk, Ilona Gurgul, Maria Oszajca, Justyna Polaczek, Konrad Kieca, Ewelina Bieszczad-Żak, Tobiasz Martyka, Grażyna Stochel

**Affiliations:** 1 Faculty of Chemistry, Jagiellonian University, 30-387 Krakow, Poland; olga.mazuryk@uj.edu.pl (O.M.); ilona.gurgul@uj.edu.pl (I.G.); justyna.polaczek@uj.edu.pl (J.P.); konrad.kieca@doctoral.uj.edu.pl (K.K.); ewelina.bieszczad-zak@doctoral.uj.edu.pl (E.B.-Ż.); tobiasz.martyka@doctoral.uj.edu.pl (T.M.); 2 Doctoral School of Science and Life Sciences, Jagiellonian University, 30-348 Krakow, Poland

**Keywords:** nitric oxide (NO), age-related diseases, NO signaling, sensing

## Abstract

Nitric oxide (NO) is a key signaling molecule involved in numerous physiological and pathological processes within the human body. This review specifically examines the involvement of NO in age-related diseases, focusing on the cardiovascular, nervous, and immune systems. The discussion delves into the mechanisms of NO signaling in these diseases, emphasizing the post-translational modifications of involved proteins, such as S-nitrosation and nitration. The review also covers the dual nature of NO, highlighting both its protective and harmful effects, determined by concentration, location, and timing. Additionally, potential therapies that modulate NO signaling, including the use of NO donors and nitric oxide synthases (NOSs) inhibitors in the treatment of cardiovascular, neurodegenerative, and oncological diseases, are analyzed. Particular attention is paid to the methods for the determination of NO and its derivatives in the context of illness diagnosis and monitoring. The review underscores the complexity and dual role of NO in maintaining cellular balance and suggests areas for future research in developing new therapeutic strategies.

## 1. Introduction

Nitric oxide (NO) is a small, highly reactive molecule that plays a crucial role in numerous physiological processes in the cardiovascular and nervous systems as a versatile signaling molecule. It its primarily synthesized endogenously by a family of enzymes known as nitric oxide synthases (NOSs), which convert L-arginine and molecular oxygen into NO and L-citrulline. In order to maintain their activity, they require several cofactors such as NADPH, FAD, FMN, and tetrahydrobiopterin (BH_4_). The NOS enzyme family exists in three isoforms: neuronal (nNOS), endothelial (eNOS), and inducible (iNOS), each with distinct roles and regulatory mechanisms [1]. It has long been believed that free NO produced by NOS acts by permeating cell membranes due to its lipophilicity and then binds to the heme moiety of soluble guanylyl cyclase (sGC) in target cells. Upon the activation of sGC by NO, it initiates the conversion of guanosine triphosphate (GTP) into cyclic guanosine monophosphate (cGMP). The increase in cGMP levels leads to the activation of protein kinase G (PKG), which, in turn, induces smooth muscle relaxation, vasodilation, and other downstream effects. This NO–sGC–cGMP pathway is fundamental to the regulation of vascular tone and blood pressure. However, recent research by Kleschyov et al. has challenged the conventional view of NO signaling. Kleschyov proposed a hypothesis and later provided evidence that NO-ferroheme, rather than free NO, serves as a key signaling entity within the vasculature [2,3]. Unlike free NO, which is rapidly scavenged by hemoglobin and other reactive molecules, NO-ferroheme is a more stable and mobile complex that can directly activate sGC without the need for free NO diffusion. This paradigm shift suggests that NO-ferroheme can be transported between proteins, partition into cellular membranes [3], and sustain bioactivity even in the presence of NO scavenger.

Due to the dual role of NO, its low physiological levels are involved in maintaining cell proliferation, differentiation, and migration, while abnormally high NO concentrations lead to an inflammatory response, growth arrest, and ultimately, cell death. Post-translational modifications of proteins including S-nitrosation, nitrosylation, and nitrosation affect a variety of cellular processes, from cGMP signaling to cell death, and tightly regulate or disrupt overall cellular homeostasis. Discussing the role of NO according to its concentration, localization, and time of occurrence will provide a better understanding of the overall importance of NO signaling in the context of various disease states and will also help in the development of new drugs that target specific diseases mediated by disrupted NO signaling.

The aging process is a complex biological phenomenon that progressively reduces tissue function and increases the risk of various age-related diseases, including heart and lung disease, diabetes, cognitive decline, and cancer. Senescent cells secrete pro-inflammatory cytokines, chemokines, and proteases, collectively known as the age-related secretory phenotype (SASP), which promote inflammation and tissue damage [4]. NO is a key regulator of many cellular activities, and its role in cellular aging and senescence has become a significant focus of research. New evidence suggests that genetic variations in nitric oxide synthases, such as nNOS and eNOS, are associated with aging and longevity, affecting cognitive and physical abilities in seniors. Decreased NO production, often caused by endothelial dysfunction, is a hallmark of aging, weakening cellular defences against apoptosis and increasing cellular vulnerability [5]. In addition, the accumulation of nitrosative and oxidative damage, particularly in mitochondrial proteins, disrupts energy metabolism and contributes to neurodegenerative diseases such as Parkinson’s disease. Understanding the complex interplay between NO signaling, cellular aging, and age-related disorders may lead to novel therapeutic strategies that modulate NO pathways to mitigate the effects of aging and improve health.

In this review, we provide a brief overview of nitric oxide signaling in various biological systems, highlighting its regulatory functions. We explore how imbalances in NO signaling may lead to its second role as a precursor of nitrogen stress and a contributor to many diseases. Additionally, we explore potential therapeutic approaches targeting NO dysregulation and discuss the role of NO in aging. Furthermore, we review current methodologies for NO detection in the NO-related pathologies, with a focus on diagnostic techniques for age-related diseases.

## 2. Nitric Oxide Signaling across Systems

### 2.1. Nitric Oxide Signaling in Cardiovascular System

NO is involved in many significant biological functions in cardiovascular physiology [6]. All existing structural forms of NOS are extensively involved in the signaling of blood vessel and cardiac cells in both physiological and pathological processes. In the vessel wall, NO is synthesized by eNOS. NO keeps vascular integrity, inhibits platelet aggregation and thrombosis, and influences vascular smooth muscle cell (VSMC) relaxation and proliferation, leukocyte–endothelial adhesion, and angiogenesis [7]. NO adjusts the activity of the cardiovascular system via two different pathways. In the direct one, NO activates sGC and causes the downstream stimulation of protein kinase G (PKG), while in the indirect pathway, NO signaling occurs due to the S-nitrosation of proteins [7,8]. Red blood cells (RBCs) are crucial in the adjustment of vascular haemostasis because they can transport oxygen between respiratory surfaces. These cells also express functional NOS (resembling an endothelial isoform) and can endogenously produce NO [9,10]. Additionally, they influence the vascular biology of NO and serve as a converter of NO bioactivity [11]. The NO diffusing from the endothelium is absorbed and inactivated by the RBC as a result of a rapid reaction with oxyhaemoglobin with the formation of methaemoglobin and nitrate, reducing the amount of NO available for vasodilation [12]. Obtaining NO by reducing nitrates and nitrites is an additional pathway to traditional vasodilation caused by NOS. Dietary nitrates undergo reduction to nitrites primarily by the human commensal oral microbiome under the tongue or in the stomach, followed by the further conversion of nitrite to NO [13]. This reduction of nitrite is enabled by proteins containing heme and molybdenum cofactors [14]. The formation of NO from nitrite by enzymatic reduction using deoxyhemoglobin could also be an optional way of NO bioactivity manufacturing and exporting by RBCs [6,15,16]. Nitrites are able to generate NO via deoxyhaemoglobin or xanthine oxidoreductase (XOR), as well as by spontaneous and carbonic anhydrase-assisted disproportionation [12].

NO produced by NOSs is involved in protecting against the initiation of both vascular and cardiac diseases in humans [7]. There are several, probably synergistic, mechanisms by which NO protects the myocardium in the course of the ischemia-reperfusion (IR) process: the reduction of Ca^2+^ overload by inhibition of L-type calcium channels (LTCC), reduction of ROS generation by mitochondrial cytochrome-c oxidase [17], and reduction of oxidative stress through the inhibition of XOR [7]. Other effects caused by NO may also contribute to the protective effect, such as coronary vasodilation, peroxide scavenging, and arginase inhibition. In recent years, the knowledge about the contribution of NOS in controlling heart function by affecting Ca^2+^ homeostasis, the sensitivity of the Ca^2+^ sarcomeric protein, and mitochondrial respiration has been enhanced [7]. The cardiac functions of eNOS and nNOS are highly dependent on their subcellular localization. In a healthy myocardium, nitric oxide generated by eNOS activation improves the relaxation of cardiomyocytes and ventricles. This is related to the lower level of intracellular Ca^2+^, through the inhibition of LTCC and increased Ca^2+^ reuptake into the sarcoplasmic reticulum (SR). The activation of the sGC–cGMP–PKG pathway leads to myosin-binding protein C (MYBPC) and troponin I phosphorylation, and subsequent reduction in myofilament Ca^2+^ sensitivity [6,7]. The nNOS-dependent modification of Ca^2+^-handling proteins in the SR is involved in reducing cytosolic Ca^2+^ concentration via phospholamban phosphorylation [7]. The involvement of NO in contractility and reaction to β-adrenergic stimulation is more complicated and not fully understood. The in vivo studies demonstrated that NO located in caveolae near the sarcolemmal LTCC blocks Ca^2+^ influx by the inhibition of β_1_-adrenergic stimulation [6,7,18]. The increased translocation of nNOS into caveolae limits Ca^2+^ flow via the plasmalemmal LTCC and can be seen as a compensatory adaptation [7]. The individuality of S-nitrosation on jointly located target proteins is also determined by NOS subcellular compartmentation. Cytosolic and sarcolemmal proteins such as LTCC and dihydrofolate reductase (DHFR) in cardiac myocytes are prioritized for nitrosation by eNOS. The transformation of dihydrobiopterin (BH_2_) to the cofactor tetrahydrobiopterin, catalyzed by DHFR, modulates the bioavailability of NOS. SR proteins such as the ryanodine receptor (RyR) are targets for nNOS located in the SR. nNOS stimulates the release of Ca^2+^ through the RyR and increases contractility, perhaps by the S-nitrosation of the RyR thiol groups [6,7]. The reversible inhibition of mitochondrial respiration by competing with oxygen in the heme iron of complex IV in the electron transport chain is another significant contribution of eNOS-derived NO to cardiac function [19].

#### 2.1.1. NOS–NO Signaling Dysfunction

The dysregulation of NOS–NO signaling occurs by two primarily important mechanisms: nitrosative stress (mostly because of ONOO^−^ generation) and the oxidative inactivation of the elements of NOS signaling [7,20]. In pathological situations, there is a decrease in the activation of the sGC–cGMP–PKG pathway due to the oxidation of tetrahydrobiopterin (BH_4_) to dihydrobiopterin (BH_2_) and sGC ferric heme, as well as NOS uncoupling [21]. The oxidation of heme iron in sGC reduces NO binding and desensitizes this protein to NO. This mechanism has been noticed in coronary heart disease, myocardial infarction, and stroke [22,23]. The function of NADH-cytochrome b5 reductase 3 (CYB5R3) in the circulatory system is to regulate the sensitivity of sGC to NO by reducing Fe^3+^ to Fe^2+^. Studies in rats have shown that CYB5R3 inhibition is associated with impaired vasodilatation and, thus, increases blood pressure [24]. The desensitization of the sGC to NO may also occur due to the S-nitrosation of the enzyme specifically at Cys122 [8]. Endothelial dysfunction, caused by oxidative stress, enhanced permeability, and leukocyte extravasation, contributes the onset of atherosclerosis. Thrombosis, platelet aggregation, as well as vasoconstriction are the result of the lower bioavailability of NO [8]. Dysregulated NOS activity is also involved in the pathophysiology of heart failure (HF) [25].

#### 2.1.2. Therapeutic Strategies to Restore NO Disfunction

The restoration of physiological NO signaling is possible by either re-establishing NO production and bioavailability or attacking components of the NO generation pathway [8]. Therapeutic strategies aimed at restoring NO bioavailability include increasing signaling by using β_3_-adrenergic receptor agonists, which would be able to avert cardiac remodeling, persist myocardial perfusion, and upgrade systolic and diastolic function [26,27]. A second type of compounds that stop endogenous inhibitory pathways and increase NO bioavailability is NADPH oxidase (NOX) inhibitors, which lessen the generation of superoxides (O_2_^−•^), thus restricting the uptake of NO and the production of peroxynitrite (ONOO^−^) [28]. Another strategy to increase the activity of NOS is based on the usage of an compounds like L-arginine and L-citrulline [29,30], arginase inhibitor [31], hydrogen sulfide [32], BH_4_ [33], and folate [34]. Folate improves DHFR activity and promotes the reductive recycling of BH_4_ [35]. NOS–NO signaling can be improved as a consequence of enhanced sGC activity. The use of vericiguat, a novel oral sGC stimulator, resulted in a reduction in cardiovascular death and hospitalisation for high-risk HF patients [36]. The elevation of cGMP levels can also be obtained through phosphodiesterase type 5 (PDE5) inhibition [37]. Those developments in therapeutic strategies to modulate NO signaling proved to be successful in preliminary and model studies, and some of them regarding to β_3_-adrenergic receptor agonists, hydrogen sulfide, improved sGC activity, and PDE5 inhibition advanced to clinical trials, which were carefully reviewed by Farah et al. [7].

Additionally, NO may be directly breathed in [38] and supplied from organic nitrates and inorganic nitrites [39]. One example is research in a murine model of a myocardial infarction, which revealed that NO inhalation reduces infraction and improves left ventricular function after ischemia and reperfusion [38]. The effect of inhaled nitric oxide has also been studied in the context of improving microcirculation in patients with severe sepsis; however, in this case, no positive effect of breathable NO on the microcirculation of blood and organ dysfunction has been found [40]. The generation of NO from nitrite is enhanced during IR; therefore, the manipulation of the NO amount in the vessels by supplementation with nitrites and nitrates is recognized as a therapeutic strategy in various pathological conditions. This reducing pathway is not dependent on NOS, and its activity increases during hypoxia and ischemia, in both physiological and pathological cases [14,41,42]. Many studies suggest that beetroot juice, with its significant nitrate content, may effectively lower blood pressure in healthy and hypertensive individuals via the nitrate/nitrite/nitric oxide pathway, making it a cost-effective strategy to reduce cardiovascular risk [43]. However, a 2022 clinical trial found no significant effect of beetroot juice on blood pressure, indicating the results may depend on dosage and treatment protocols [44]. Using animal models, nitrates have been shown to be protective in IR injuries and some other cardiovascular diseases [6,41]. Furthermore, the delivery of nitrates in clinical trials had an advantageous impact on blood pressure and general vascular condition [41]. The primary goal and the main difficulty are finding the optimal space and time distribution of potential therapeutics to obtain the intended effects while not inducing undesirable disruptions in physiological NO signaling. Moreover, the use of organic nitrates as therapeutics is restricted by unfavorable pharmacokinetics and the development of tolerance [45]. The therapeutic strategies targeting NO signaling and dysfunction across various diseases are summarized in Table 1.

### 2.2. Nitric Oxide Signaling in Central Nervous System

**Figure 1:**
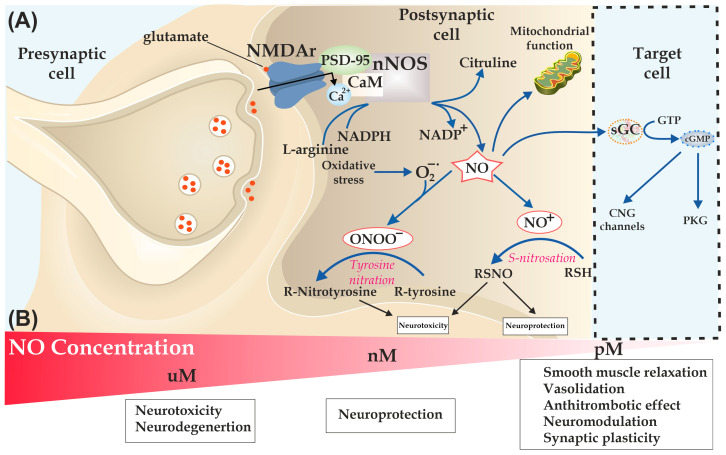
(**A**) General nitric oxide signaling pathways in physiology and pathology. (**B**) NO neuroprotective or neurotoxic effects depending on its concentration and the cellular environment. A low and transient NO level has neuroprotective effects. A high level of intercellular NO in the presence of oxidative stress leads to neurotoxic and proapoptotic effects.

Nitric oxide is a significant signaling molecule that has a crucial role in physiological neuronal and synaptic function [63]. The effects triggered by NO depend on its concentration (Figure 1B). Although a physiological level of NO is essential for signal transmission and neuroprotection, the overproduction of NO mediated by oxidative stress results in neuroinflammation and neurodegenerative disorders [64]. In the central nervous system (CNS), NO production is mediated by nNOS, which requires the calcium (Ca^2+^)/calmodulin (CaM) complex for its activation (Figure 1A). The Ca^2+^ flow occurs through N-methyl-D-aspartate receptors (NMDAr) [65]. nNOS is the predominant isoform of nitric synthases in the CNS, occurring in almost all brain areas, however, with a certain advantage in the olfactory bulb, the cerebellar cortex, the striatum, the hippocampus, and the hypothalamus. Additionally, nNOS occurs mainly in glutaminergic and GABAergic neurons [6].

NO can cause neuroprotective or neurotoxic effects depending on its concentration and cellular environment. At a low level of NO, its main impact is directed at iron-heme proteins. NO can influence neuronal activity and excitability by activating the canonical sGC/cGMP-dependent pathway [66]. The sGC/cGMP pathway has a key role in controlling cerebral blood flow, by modulating smooth muscle relaxation, and mediating the modulation of synaptic neurotransmission and plasticity. The cGMP-triggered activation of the cAMP response element binding protein (CREB) or protein kinase G (PKG) may have a neuroprotective effect. This effect can also occur due to the S-nitrosation of specific target proteins, resulting in either the inhibition of proteins (NMDA receptor [NMDAR] and caspase 3) or their activation (hypoxia-induced factor 1 [HIF-1]), heme oxygenase 1 [HO-1], and histone deacetylase 2 [HDAC2]) [63,67]. Excessive NO production, particularly in the setting of oxidative stress, which causes the production of ONOO^−^, triggers the S-nitrosation or nitration of critical proteins in the cytosol and mitochondria, promoting cell injury via protein misfolding, mitochondrial dysfunction, synaptic injury, and apoptosis [63]. The various functions of NO in neuronal communication can be explained by the expression of nNOS in both excitatory and inhibitory neurones, as shown by several in vivo studies using mice with a defective nNOS gene [6].

Neurovascular coupling (NVC) is a mechanism that supports the coupling among cerebral blood flow (CBF) and neuronal activity. Nitric oxide is considered as the most significant molecule in this process, among other vasoactive molecules released by glutamatergic activation, and is necessary for the neurovascular response to occur [66]. An association has been found between NVC malfunction and neurodegeneration, and cerebral vascular hypoperfusion is involved in the pathogenesis of many neurodegenerative disorders. NVC involves a biphasic enhance in CBF to meet the metabolic demand. A recent analysis of preclinical studies showed that up to two-thirds of NVC responses are related to nitric oxide signaling mechanisms [68]. Oxidative stress reduces NO-mediated CBF and tends to reduce NO bioavailability. Peroxynitrite production is also one of the reasons NVC does not work properly [67]. Hoiland et al. investigated the pathways through which NO signaling participates in NVC in humans. A competitive non-selective inhibitor of NOS L-NMMA was found to reduce NVC response by 30%, which confirms the crucial role of NO in human NVC [66].

#### 2.2.1. Nitric Oxide Dependent Post-Translational Modifications—Role in Neurodegenerative Diseases

The high level of intercellular NO in the presence of oxidative stress leads to neurotoxic and pro-apoptotic effects, leading to the development of neurodegenerative disorders [63]. Abnormal NO production may be an effect of the activation of nNOS through NMDA receptors or the induction of iNOS activity by pro-inflammatory cytokines [63]. This contributes to the development of pathological conditions like stroke, epilepsy, and neurodegenerative disorders by protein S-nitrosation or the nitration of tyrosine residues [6,66]. The regulation of NMDAR activity is of paramount important for the correct operation of the brain and the avoidance of brain damage.

The rapid reaction of NO with O_2_^−•^ leading to the formation of ONOO^−^ is the main pathway of the deleterious action of NO [67]. The formed ONOO^−^ causes the nitration of protein tyrosine residues (nitrotyrosylation); this produces 3-nitrotyrosine (3-NT), a chemical determinant of both nitrosative and oxidative stress [63]. Protein tyrosine residue nitration is considered as a crucial element in the etiology of neurodegenerative disease [64]. The pathogenesis of neurodegenerative disorders is related to neuroinflammation, as well as the aggregation of misfolded proteins such as α-synuclein in Parkinson (PD) patients and amyloid β peptide (Aβ) and tau in Alzheimer (AD) patients. Abnormal protein aggregates were found in the neuronal bodies of the limbic and cortical areas of the brain in PD [69,70]. These effects are often assigned to anomalous RNS/ROS production [71]. Extensively nitrated tyrosine in α-synuclein promotes its oligomerization, and hence, the formation of Lewy bodies. This is also related to the autophagic reaction of blood cells in patients with idiopathic PD [72] and activated neuroinflammation, which subsequently results in excessive RNS production [73]. AD, the most commonly occurring dementia, is also associated with tyrosine nitration [71]. Studies indicate the tau protein is nitrated, which causes its greater aggregation [67]. The nitration and oxidation of other important proteins such as the mitochondrial isoform of superoxide dismutase (SOD) has been confirmed [74,75]. A large amount of inactivated by nitration glyceralaldehyde-3-phosphate dehydrogenase (GAPDH) was found in the hippocampus of AD patients. It can cause the consumption of adenosine triphosphate. Improper GAPDH encourages the aggregation of insoluble proteins causing cell death [70]. Furthermore, peroxynitrite is an important mediator of lipid peroxidation and mitochondrial dysfunction, leading to DNA damage, which finally leads to apoptosis and necrosis [76,77].

NO can combine with oxidant molecules to form RNS that elicit post-translational modifications such as S-nitrosation (RSNO) [63]. Under a basal level of nitrosative stress, a subgroup of proteins is S-nitrosated, supporting cell signaling cascades to protect the nervous system. When RNS levels are increased, the S-nitrosation of additional protein can bring misfolding, neuronal and synaptic injuring, the deregulation of mitochondrial function, and apoptotic cell death [66]. Studies of S-nitrosated proteins in the brain tissues of patients with AD, PD, and other disorders provide powerful arguments that this modification is associated with the pathogenesis of the disease [63,78]. An example is Hsp90: Its S-nitrosation causes loss of its chaperone functions and promotes the accumulation of β-amyloid and tau aggregates [79]. In the course of AD, the S-nitrosation of proteins affects mitochondrial homeostasis and metabolism. One of the proteins that is abnormally S-nitrosated in AD is a neuronal cyclin-dependent kinase (CDK5). The S-nitrosationed CDK5 transnitrosylates mitochondrial-fission protein DRP1, triggering the loss of dendritic spines and neuronal injury [78]. The S-nitrosation of voltage-dependent anion-selective channel protein 2 (VDAC2) inhibits its function, similar to the effect observed with VDAC1, leading to impaired calcium ion influx into the mitochondria. Additionally, the S-nitrosation of VDAC2 disrupts microtubule architecture, resulting in neuronal injury [78,80]. Pro2 is another mitochondrial protein that is recognized as abnormally S-nitrosated in the AD. The modification of this protein impairs its detoxifying activity [81]. In PD, one of the identified S-nitrosated proteins is parkin, the S-nitrosation of which markedly diminishes its E3 ligase activity and protective function [78,82]. The S-nitrosation of parkin also suppresses its inhibitory effect on mitochondrial fission protein DRP1 activation, resulting in increased mitophagy and neuronal damage. Furthermore, it undermines its interaction with PTEN induced kinase 1 (PINK1), a putative mitochondrial serine/threonine kinase that preserves cells from ROS-induced apoptotic cell death, preventing damaged mitochondrial clearance. Mitochondrial proteins that are S-nitrosated in PD are prohibitin and peroxiredoxin 2. Modifications of these proteins inhibit their antioxidant activities [78].

The homeostasis of divalent redox metal is of great importance in cell redox status determination. Numerous studies have shown that metals are misregulated in neurodegenerative disorders [66,83,84]. In the brain, iron plays a key role in some physiological functions, such as DNA synthesis, neurotransmitter metabolism, oxygen transport, and mitochondrial respiration. Iron homeostasis is closely related to the neurodegenerative process through NO overproduction and related to its ONOO^−^ production. Aβ plaques, similarly to tau tangles, contain significantly more iron in people with AD [76,85], as well as the substantia nigra of PD patients [86]. NO can interact with proteins that contain [Fe-S] clusters, for example, iron regulatory protein (IRP), and by this, influence their enzyme activity [76]. The interaction with NO influences the binding of IRP to iron responsive element (IRE), leading to modifications in the transcription of iron-metabolism-related proteins. The inhibition of ferritin, protein, and amyloid-β precursor protein (APP), as well as the activation of transferrin receptor proteins (TfR) and divalent metal transporter 1 (DMT1), causes an increase in intercellular iron levels. NO also influences iron homeostasis not only by the regulation of IRP-IRE binding, but also by the S-nitrosation of divalent metal transporter 1 (DMT1). The S-nitrosation of DMT1 enhances its activity, further contributing to an increase in intracellular iron levels [76]. The homeostasis of metals is also associated with neurodegenerative diseases. Both very high and low levels of zinc are linked with the greater production of ROS and RNS. A decreased zinc level has been observed in the serum of AD patients and has been connected to the greater deposition of Aβ in the brain.

#### 2.2.2. Therapeutic Targets in Neurodegenerative Disease

The significant role of NO in the nervous system can result in the incorrect regulation of NO, leading to various pathologies. Since the excessive activation of nNOS causes high intracellular levels of nitrites and superoxides, which ultimately generate RNS, including peroxynitrates, nNOS inhibitors are potential drug candidates for patients with neurodegenerative disorders. The early tested inhibitors of NOS were arginine analogues. Unfortunately, these types of compounds have shown poor selectivity, which is important due to the significant role of eNOS in vascular regulation. Since the inhibition of the wrong isoform of NOSs can cause undesirable side effects, many attempts have been made to target nNOS selectively among the remaining isoforms. The identification of the nNOS crystal structure was the driving force behind this [87]. Selective inhibition has been attempted using various approaches, such as competitive inhibition (inhibition by mimicking a substrate or cofactor), dimer inhibition, and CaM antagonism (preventing the reaction between the enzyme and regulatory proteins). Specific examples of nNOS inhibitors are discussed in detail by Mukherjee et al. [46]. Another perspective therapeutic approach for many neurodegenerative disorders is the regulation of protein S-nitrosation. An example is nitromemantine, a low-affinity voltage-dependent noncompetitive antagonist in glutamatergic NMDA receptors. Nitromemantine causes a significant increase in the S-nitrosation of the GluN1 subunit of the NMDAR [47]. Another potential drug named CGP3466b blocks GAPDH nitrosation with no effect on enzyme activity, producing an antiapoptotic effect. Recent studies indicate that SNO-GAPDH is involved in neuro-axonal damage in neuroinflammation and recognize CGP3466B as a potential neuroprotective agent in the treatment of multiple sclerosis [48]. For both nitromemantine and CGP3466B, some desirable effects were observed due to the direct and specific changes in the formation of SNO-NMDAR and SNO-GAPDH, respectively. Nevertheless, more research is needed to prove that their use can be safe and effective [88].

### 2.3. Nitric Oxide Role in Immunity

Nitric oxide plays an essential role within the human immune system [89]. Macrophages, T cells, natural killer cells, and other cells of the immune system produce NO thought the expression of iNOS, utilizing it as a toxic agent against infectious particles as a part of the human immune system [90]. In response to appropriate stimuli such as bacterial lipopolysaccharides and proinflammatory cytokines, other types of cells can also express iNOS [91]. The cellular pathways leading to iNOS induction exhibit cell line-specificity. Nevertheless, in the majority of cells, the activation of transcription factors NF-κB and STAT-1a plays a pivotal role. These factors translocate to the nucleus and bind to the promoter region of the iNOS gene, which triggers the protein expression [6].

The functions of NO produced by iNOS differ from NO generated by other NO synthases (eNOS and nNOS). To be an effective anti-infectious agent, NO needs to be generated for a prolonged period at high micromolar concentrations. Calcium-independent iNOS can meet these requirements. The reactivity of NO is determined by its concentration, duration of time, and surrounding environment. Within an inflamed setting, the simultaneous presence of elevated NO levels and superoxide results in the production of a highly hazardous ONOO^−^. Produced RNS directly react with iron or thiol groups of crucial enzymes responsible for DNA replication, repair, cellular metabolism, and mitochondrial respiration [6,92]. The nitration of essential proteins, including cytochrome c, fibrinogen, and MnSOD, has been reported both in vitro and in vivo [93]. Other examples of relevant reactions include the inhibition of DNA synthesis due to Zn release via the S-nitrosation of Zn-containing metalloproteins [94] or the inhibition of respiration due to the S-nitrosation of critical thiols in mitochondrial complex I or GAPDH [95,96]. By interfering with such diverse signaling pathways, produced RNS can successfully suppress pathogen replication [97]. The antimicrobial activity of NO has been reported against bacteria, fungi, and even viruses, including the recent medical challenge of the SARS-CoV-2 virus [98,99]. High NO levels have been utilized in anticancer therapy to exert antitumor effects [100,101]. The protective role of NO is visible in the reports regarding the constitutive expression of iNOS in human lung and paranasal epithelial cells [102,103]. The expression of iNOS in the upper airways is integral to innate immunity, playing a vital role in preserving barrier integrity and safeguarding against pathogen infiltration.

Although most studies focus on the immunological role of iNOS, the activity of eNOS has also been implicated in immune regulation [104,105]. NO from eNOS is involved in T cell activation via the S-nitrosation of Cys374 in β-actin [106]. In T cells, the activation of eNOS further modulates the production of inflammatory cytokines [105]. When present in low concentrations, NO selectively triggers the activation of sGC, leading to the induction of interferon γ (IFN-γ) expression, but the reduction of the production of interleukin 2 (IL-2) [105,107]. IFN-γ strongly stimulates macrophages to produce high concentrations of NO via the activation of iNOS [107]. This indicates that eNOS might have a crucial function during the initial stage of immune responses before the involvement of iNOS-expressing cells. However, the overall impact of eNOS in immunoregulation has not yet been fully clarified, as various studies regarding the protective vs. disease-exacerbating functions of eNOS have been reported [108,109,110].

The overall role of NO in immunity is not only anti-inflammatory [6,92]. A high concentration of NO is also linked to its immunosuppressive activity [111]. At high levels, NO causes increased p53 molecule activity due to its phosphorylation and stabilization [112]. In the immune system, p53 blocks lymphocyte proliferation and causes macrophage apoptosis [111]. Additionally, at high dosages, NO blocks interleukin 12 (IL-12) signaling by activated macrophages, which suppresses T cell functions [113,114]. This feedback mechanism illustrates the fine-tuning of the immune system by NO. At the early stage of the infection, low levels of NO increase the development of T cells, which help to control infection development [107]. As a result of macrophage activation by IFN-γ, T cells would subsequently enhance the levels of NO, resulting in the production of substantial amounts of NO that possess cytotoxic activity. However, the excessive activation of T cells is associated with different immunopathologies. The selective inhibition of T cell development and differentiation occurs in the presence of elevated levels of NO, while established T cells remain unaffected by its effects [113]. The generation of NO by iNOS in activated T cells further impairs interleukin-17 (IL-17)-producing T cells through the tyrosine nitration of the transcription factor RORyt [115]. This way, NO elegantly regulates the intensity of the immune response.

Several studies have implicated the involvement of NO in acute and chronic inflammation. NO is associated with several known immunopathologies, including rheumatoid arthritis, septic shock, and systemic lupus erythematosus [107]. Excessive NO levels are reported in some viral diseases, including H5N1, the 1918 influenza viruses [116], and HIV [117,118]. Reduced morbidity and mortality and a beneficial survival effect were observed during in vivo studies of highly pathological influenza viruses after the systemic use of NOS inhibitors in the groups of knock-out mice iNOS−/− [116,119]. NO produced from iNOS is associated with the severity of ulcerative colitis in dogs [120] and plays a role in the onset and persistence of inflammatory bowel disease [121]. Even cancer-related pain is associated with the upregulation of iNOS and nNOS [122]. Various theories exist about the role of iNOS during sepsis [123]. There is evidence suggesting the involvement of iNOS in the pathogenesis of septic shock [124]. Its mechanism of action rests on vascular collapse due to the massive amounts of NO and substantial overwhelming sGC activation. Nevertheless, contrary findings from other in vivo studies have emerged, indicating no significant influence of iNOS on the survival rate of mice in the septic shock model [125].

Given the numerous reports on the pathological role of NO overproduction in the immune system, blocking NO production or signaling could have considerable therapeutic benefits. Some in vivo studies have indicated that autoimmune diseases are delayed or suppressed by the iNOS inhibitors’ administration [49,89]. Others report no improvement in allergic encephalitis, autoimmune encephalomyelitis, or multiple sclerosis [55]. A variety of specific and non-specific inhibitors targeting iNOS have been created and effectively evaluated in preclinical models concentrating on systemic inflammation and pain [49,126]. Some of them even reached clinical trials, but failed. As of now, there are no approved clinical iNOS inhibitors available [6,49]. The reason for that is probably an ambiguous role of NO in the inflammatory process that is too complicated to simply “inhibit NO production.” Several clinical trials have explored the use of inhaled NO as a treatment strategy in COVID-19 patients, aiming to lower the frequency of mechanical ventilation and intubation, improve oxygenation, and achieve other therapeutic benefits [50,51]. The therapeutic potential of NO donors (GSNO, SNAP, SNP, and others) in the treatment of autoimmune disease, inflammation, and stroke has also been evaluated. Some experiments revealed that the administration of GSNO provided an anti-inflammatory effect by reducing proinflammatory cytokine synthesis and endothelial–leukocyte interactions through the inhibition of the NF-κB pathway due to its S-nitrosation [52,53,54]. The beneficial effect of NO produced by UV irradiation was reported in human atopic dermatitis [55,56]. While no NO-based immunotherapies have been introduced yet to treat inflammation or immune diseases, the possibility of their introduction in the future is promising.

### 2.4. The Dual Role of NO in Cancer

**Figure 2:**
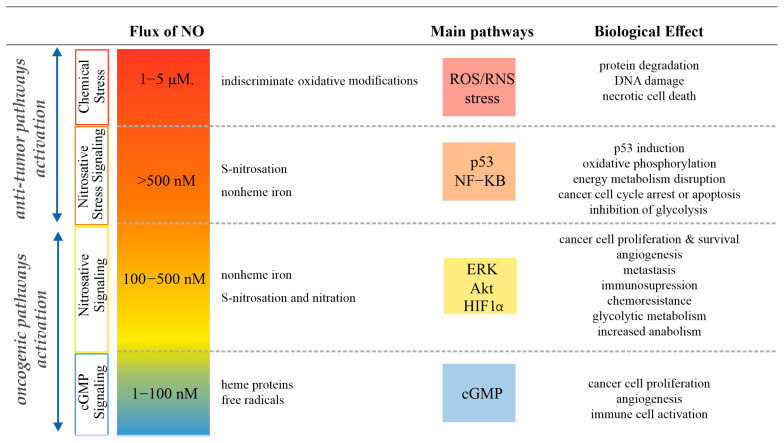
Pro- and antitumorigenic NO signaling.

The dual nature of NO, with both pro- and anticancer properties, presents challenges in developing NO-based therapies and unraveling NO-dependent signaling pathways. The biological impact of NO on cancer greatly relies on factors such as its concentration, localization, and duration of exposure. Considering the involvement of NO in various cancer-related processes, including tumor growth, invasion, migration, metastasis, and angiogenesis, even slight alterations in its temporal and spatial concentration can have important consequences. At low concentrations, NO is involved in protumorigenic mechanisms, while high levels result in cancer cell death. The elevated expression of NOSs has been observed in diverse epithelial tumors, including breast, lung, ovarian, prostate, colorectal, and other cancer types, indicating the participation of NOSs in cancer cell signaling pathways. Furthermore, iNOS has been reported as a negative prognostic factor in various cancers [127,128]. Inflammation, which often coexists with cancer, can lead to high levels of activated macrophages that produce NO. This alteration of signaling pathways plays a pivotal role in the accelerated progression of cancer compared to healthy tissue. To investigate the intricate biology of NO, exogenous NO donors known as amine-based diazeniumdiolates, commonly referred to as NONOates, serve as valuable tools for studying the chemical and molecular mechanisms involved. These compounds spontaneously generate NO and mimic NO fluxes produced by NOS. Depending on the concentration of NO, four different mechanisms of NO action in cancer have been recognized (Figure 2).

When NO levels are low (<100 nM), NO-induced actions are mediated through direct interaction via the nitrosylation of the heme group of sGC, which subsequently triggers the synthesis of cGMP. Normal physiological levels of NO may be dysregulated by different cancer-related processes. cGMP is involved in numerous biological processes, such as angiogenesis and neovascularization, which are important in terms of cancer initiation and progression. Kinetics studies revealed that in hypoxic solid tumors, NO is most likely generated by constitutive eNOS [129,130]. K.-H. Lim et al. showed that eNOS can directly activate the oncogenic Ras family of proteins via S-nitrosation at cysteine 118 [131]. This process of nitrosation is correlated with an elevation in enzyme activity and is essential in tumorigenic mechanisms [131,132,133].

The second level of NO (100–500 nM) is often referred to as nitrosative signaling. At this NO level, the activation of specific cellular signaling leads to an increase in the genomic instability, angiogenesis, proliferation, metastasis, as well as chemoresistance and immunosuppression in aggressive tumors [134]. Signaling within this NO level occurs though direct interactions, although with slow kinetics, involving nonheme iron and the nitrosation of essential thiol groups in membrane proteins [135]. Due to the higher NO and O_2_ solubility in lipids, the signaling events occur preferentially in membranes and do not influence the downstream signaling reactions [134]. At this concentration, NO acts as a hypoxia mimicking agent by stabilizing hypoxia-inducible factor HIF1α [136]. This stabilization occurs through the inhibition of prolyl hydroxylases (PHD), which are Fe^2+^-dependent enzymes responsible for HIF1α degradation [137,138]. Consequently, hypoxia responsive elements (HREs) are activated [139], leading to the activation of various proteins involved in metabolic signaling. Thus, NO induces a hypoxia-like state even when there is an adequate oxygen level present [134]. PHD-like enzymes not only are involved in the inhibition of HIF1α activity, but also are capable of influencing the NF-κB pathway that is important in the promotion and progression of tumor [140]. Iron metabolism at this NO level is also regulated by the reaction of NO with other nonheme iron proteins, like iron regulatory protein 1 (IRP1). NO stimulates the activity of IRP1 by directly targeting its [4Fe-4S] cluster, promoting the cluster’s gradual disassembly and complete removal, which increases iron uptake and decreases its secretion [141,142,143]. Ras and epidermal growth factor receptor (EGFR) might also be activated though RNS-mediated nitrosation though the ligand-independent pathway in contrast with a lower level of NO. Such nitrosation leads to the activation of the RAF/MEK/ERK pathway, which transports a signal of cellular surface to the DNA in the nucleus. Ras activity can further be modulated by S-glutathionylation at this NO level [134]. The S-nitrosation of another important group of enzymes matrix metalloproteinases (MMPs) also greatly affects their function. The nitrosation of cysteine residues in MMP9 releases Zn^2+^ ion, which is present in the enzyme active site, activating MMP zymogen and, thus, promoting tumor growth and angiogenesis [144,145]. Nitrative and oxidative modifications may also take part in the signaling at this NO level. Tissue inhibitor of matrix metalloproteinases 1 (TIMP1) is the protein responsible for the proteolytic activity of matrix metalloproteinases (MMPs). At nitrosative signaling, the nitration of TIMP1 is observed, which not only decreases its ability to inhibit MMPs’ activity, but also activates pro-survival PI3k/Akt/BAD signaling [146]. Generally, at this NO level, the activation of Akt, one of the critical proteins in many protumorigenic pathways, is observed even in the absence of growth factors and associated ligands.

The next NO level is called nitrosative stress signaling and is defined by sustained NO fluxes between 500 and 1000 nM. At this level, more indiscriminate nitrosation occurs. This concentration of NO is involved in both antitumor (pro-apoptotic and cytotoxic) and protumor (anti-apoptotic and pro-survival) effects [134,147]. One of the most important events that occurs at this level is the activation of p53 [148,149]. The significance of p53 in cancer becomes apparent due to the fact that it is highly mutated (>18,000 mutations) in many different types of cancer and is often deactivated by several diverse mechanisms [150]. High concentrations of NO phosphorylate and stabilize p53 [90], illustrating the NO-protective action against cellular damage due to the p53 activation of cell cycle checkpoints. This signaling pattern is indicative of the wild-type form of p53, whereas mutated p53 may be unresponsive to the growth-arresting effects induced by elevated concentrations of NO [136]. A similar tumor-suppressor effect is observed due to the S-nitrosation of another enzyme, tissue transglutaminase (TG2) [144]. The protumorigenic effect of NO is evident in various processes. The S-nitrosation of key DNA repair enzymes can modulate their activity by disrupting the zinc finger motif, thereby decreasing the protective effect of p53 activation [151]. The interaction with NO causes the loss of the zinc ion and inhibits the catalytic activity of human 8-oxoguanine glycosylase (hOGG1), which is responsible for repairing 8-oxoguanine through base excision [152]. Additionally, NO inhibits caspase activation and displays anti-apoptotic activity by reversibly S-nitrosating the cysteine residue at the caspases’ active site [149]. Finally, at this NO level, the inhibition of NF-κB signaling occurs due to the S-nitrosation of the cysteine 62 residue [153].

The fourth NO level is characterized by fluxes of NO with concentrations greater than 1 µM. These high locally generated concentrations of NO and ROS produce different forms of RNS, which induce various modifications of DNA and proteins, leading to chemical stress conditions [135]. The nitrosylation or oxidation of respiratory complex proteins is induced by high levels of NO, causing the removal of iron from the [Fe-S] clusters and resulting in mitochondrial depolarization [154]. The production of peroxynitrite due to the reaction of NO with superoxide can irreversibly inhibit the enzymatic activity of cytochrome c oxidase, inducing permanent damage to the mitochondrial respiration system and leading to pro-apoptotic changes in cellular metabolism [155]. At this level of NO, p53 does not orchestrate apoptotic cell death, but rather, activates necrotic processes. This occurs due to the accumulation of p53 in the mitochondrial matrix, which triggers the opening of the mitochondrial permeability transition pore (PTP). Through direct interaction between p53 and the PTP regulator cyclophilin D, necrotic cell death is activated [156]. Such high levels of NO occur physiologically within the macrophage microenvironment and lead to apoptosis and mitochondrial dysfunctions that are relevant in fighting different pathogens [134]. In the case of anticancer therapy, these chemical stress levels of NO play a significant role in the patient’s response to diverse immunotherapy protocols.

#### NO-Based Therapeutic Approaches for the Treatment and Prevention of Cancer

The diverse roles that NO plays in cancer biology provide unique opportunities for cancer treatment and prevention, ranging from regulating tumor growth and angiogenesis to modulating the immune system’s response to cancer cells. Sustained low levels of NO can create a favorable “zone” for the growth and promotion of cancer cells. Even a slight shift in NO levels, whether higher or lower, can disrupt the conditions necessary for cancer cell proliferation. Therefore, therapeutic anticancer strategies can include inhibiting NO biosynthesis, as well as administering exogenous NO donors, depending on the stage of cancer. Due to its involvement in various signaling processes, it is crucial to take precautions to avoid any potential adverse systemic effects when utilizing NO-based therapies.

Given the significant role of iNOS in cancer, utilizing NOS inhibitors to regulate NO levels and localization represents a promising therapeutic approach. Non-cancer human studies have already evaluated the efficacy of small molecule iNOS inhibitors, showcasing their potential for advancement into clinical trials targeting solid tumors. In preclinical human melanoma models, the iNOS inhibitor N6-(1-iminoethyl)-L-lysine dihydrochloride (L-nil) effectively suppressed melanoma growth by inhibiting NO generation both in vitro and in vivo [57]. The suppression of cancer growth is linked to a reduction in the formation of tumor microvessels, the inhibition of the antiapoptotic protein Bcl-2, and a synergistic effect with conventional cisplatin treatment. Numerous other NOS inhibitors have undergone comprehensive testing in various preclinical studies as potential therapeutics for cancer treatment [157]. The results of phase 1/2 clinical trials of another pan-NOS inhibitor L-NMMA (NG-monomethyl-L-arginine), in triple-negative breast cancer (TNBC) have recently been published [58]. Chung et al. reported enhanced chemotherapeutic response in patients with locally advanced breast cancer and metastatic TNBC when L-NMMA was combined with taxane. Additionally, L-NMMA is being evaluated in phase 1b clinical trials, in combination with the anti-PD1 humanized antibody pembrolizumab, for the treatment of melanoma, non-small lung cancer, and several other types of solid cancers [59].

The second strategy is based on providing tumor cells with elevated levels of NO. High dosage of NO can exert an anticancer effect by several different pathways, which can be broadly classified into two categories: direct cell lysis and the reversal of multidrug resistance [158]. A high concentration of NO may directly inhibit cancer cell growth through the following mechanisms: (1) upregulation of the pro-apoptotic p53 pathway; (2) permeability of mitochondria and release of cytochrome c; and (3) generation of ONOO^−,^ which induces cell cycle arrest, angiogenesis inhibition, cytotoxicity, and cell necrosis [60]. When high fluxes of NO occur, a variety of RNS can be generated, which, in turn, cause the oxidation and deamination of DNA bases, stand breaks, or cross-linking. Extensive research is currently being conducted to explore the anticancer potential of various major categories of NO donors, such as organic nitrates, diazeniumdiolates, metal-NO complexes, furozans, S-nitrosothiols, and syndonimines [60]. It is important to note that compounds that are traditionally referred as NO donors vary vastly in their pharmokinetic and signaling properties, allowing for different types of anticancer treatments [158]. Preclinical and clinical data on anticancer properties of several NO donors showed a beneficial therapeutic effect [62,158]. The drugs are able to enhance antitumor immunity and act as antihypoxic agents, modulating cellular mechanisms that promote treatment resistance and enhance survival. The immunosensitizing ability of NO donors has also been reported. The diazeniumdiolate DETANONOate sensitisized cancer cells to apoptosis though the inhibition of NF-κB pathway due to the S-nitrosation of p50 [61].

Another promising strategy in the battle against cancer is the combination of NO donors with other anticancer therapies. Numerous preclinical and clinical studies have demonstrated that NO donors have the potential to enhance the therapeutic efficacy of various anticancer agents, including cisplatin, docetaxel, and carboplatin [60,62]. NO donors usually sensitize chemo- and radioresistant cancer cells by stabilizing HIF1α, improving blood flow and oxygenation. The pretreatment of cancer cells with NO donors has the potential to enhance the activity of transcription factors like activator protein-1 (AP-1) and facilitate the translocation of NF-κB into the nucleus, leading to the increase of the transcription of iNOS [159]. This, in turn, can generate even higher levels of NO, resulting in the generation of cytotoxic levels of RNS. A synergetic effect between NO and photodynamic therapy (PDT) sensitizers has also been reported [160,161].

Given the variety of roles NO plays in human physiology, every aspect of its delivery (concentration, place, and time) should be precisely controlled. Currently, various nanodelivery systems for precise NO delivery are under development [60,158]. NO-based anticancer therapy still has some severe unsolved issues. The biological mechanism of action of NO donors and inhibitors needs additional study to fully evaluate the potential and consequences of such treatment. Taking into account recent findings regarding the involvement of NO in numerous diseases, it is crucial to thoroughly assess the risk–benefit balance of such therapies. There is also an unresolved need for efficient delivery systems that allow for full control of the release of NO. However, the treatment of cancers with NO-based drugs alone or in combination with other types of therapies has a promising future and deserves additional investigation.

### 2.5. Nitric Oxide in Clinical Studies

Several therapeutic strategies to restore NO disfunction are currently being tested in clinical trials.

The role of NO in cancer and anticancer therapeutics was summarized in a recent review by Mintz et al. [100]. The majority of strategies are based on the cytotoxic activity of NO at high concentration. Several NO donors are being tested alone or in combination with different anticancer drugs. The benefits of nitroglycerin, a known NO donor, are shown in lung, prostate, liver, colon, and brain cancers [162]. The administration of arginine, a NO precursor, is proven to enhance the effect of radiation in patients with solid tumors and brain metastasis [163]. To improve the NO-based therapy of cancer, several strategies such as the selection of a highly specific delivery system, the encapsulation of drugs, and the on/off delivery technique can be used [164]. Another reported NO-based strategy to treat cancer relied on the use of NOS inhibitors. An example is NG-monomethyl-l-arginine (L-NMMA), a known NOS inhibitor, which, when combined with taxane, showed enhanced survival in patients with triple-negative breast cancer and locally advanced breast cancer (NCT02834403, [58]). Another combination of L-NMMA with pembrolizumab is supposed to increase the response of the immune system in patients with a variety of different types of cancer (NCT03236935).

NO donors are also good candidates for acute stroke treatment [165]. The treatment with nitroglycerin in cases of acute stroke was associated with improved functional outcomes, activities of daily living, cognition, and quality of life, and fewer deaths (NCT00989716, [165]). NO administration in cases of cardiopulmonary bypass circuit during cardiac surgery was associated with fewer incidents of acute kidney injury (NCT03527381, NCT01802619, [166,167]). Several competitive inhibitors of iNOS were proven to be potent pharmacological tools in cases of stroke, hyperalgesia, renal ischemia, and reperfusion injury [168]. However, none of these compounds progressed into advanced clinical trials, mainly due to severe preclinical toxicity or few therapeutic benefits.

Inhaled NO is well documented to improve oxygenation in a variety of pathological conditions [169,170]. The use of inhaled NO has been popular during pediatric and adult cardiac surgeries and lung and heart transplantations [171]. Unfortunately, numerous clinical trials showed that the reproducible physiological benefits of inhaled NO do not translate into meaningful and prolonged clinical outcomes on morbidity and mortality. The use of inhaled NO was left as a practice-based recommendation for the perioperative management of difficulties in high-risk cardiac surgery [171].

The beneficial effects of NO inhalation during the treatment of patients with COVID-19 infection was studied in a variety of clinical trials (e.g., NCT04306393, NCT04397692, NCT04606407, NCT05430503, NCT05599919, and NCT05721144). Different NO-releasers (R-107 and COViNOX) are currently being studied in clinical trials (NCT04421508, [172]). A rapid effect on greater RNA reduction was shown with the use of nitric oxide nasal spray (NONS) in non-hospitalized adult Asian patients, where patients reached negative RT-PCR status four days earlier than the placebo group [173]. However, no randomized controlled data regarding the use of inhaled NO in COVID-19 are currently available, and published data are largely derived from retrospective and cohort studies [174]. Observed improvements in oxygenation did not always translate into significant clinical outcomes. Therefore, current clinical guidelines do not routinely recommend NO use in COVID-19 patients. The use of NO should be considered on an individual basis.

Increased interest in the scientific community is attached to the dietary supplementation of nitrate. Recently, numerous clinical studies demonstrated the benefits of beetroot juice supplementation for enhancing exercise tolerance, reducing blood pressure, decreasing inflammation (NCT04584372, [175]), protecting against cold symptoms, and reducing negative indicators of stress (NCT03159273, [176]). A single dose of microencapsulated watermelon ring rich in L-citrulline improved vascular function in young adults (NCT04781595, [177]). The supplementation of inorganic nitrate (KNO_3_) in patients with acute coronary symptoms decreased the rate of contrast-induced nephropathy (NCT03627130, [178]).

The majority of clinical trials regarding NO modulation in pathological conditions failed to show significant therapeutic benefits despite undisputable physiological benefits. Many of these trials have only been conducted with a low number of patients or failed due to inadequate study design. The efficiency of NO modulators in vivo may be insufficient to achieve therapeutic benefits in clinical trials, probably due to inappropriate safety, NOS polymorphism, or lack of tissue specificity. Another reason for the lack of success in clinical trials may be differences in disease pathomechanisms in different species, which may explain why preclinical animal models are not sufficiently predictive. Better mechanistic understanding of the role of NO is needed, as well as suitable delivery systems coupled with appropriate trial designs.

## 3. NO Signaling in Aging

Aging is a fundamental biological process that negatively affects tissue functions and increases the risk of numerous age-related conditions, including heart diseases, ischemic stroke, diabetes, dementia, and cancer. Given the involvement of NO as a signaling molecule in nearly all cellular processes, extensive research is dedicated to understanding its role in cellular senescence and the aging process. Strong evidence of NO’s involvement in the aging process has been reported. Montesanto et al. reported that gene variations in nitric oxide synthases are associated with age-related phenotypes and longevity [179]. Gene variations of nNOS and iNOS are associated with declined cognitive and physical performance. Single-nucleotide polymorphisms in nNOS and eNOS genes are also linked to quality of life among older individuals, such as occurrences of depression and disabilities.

The endothelial dysfunction that is often observed during aging is caused by the diminished availability of NO, particularly in pathological states like atherosclerosis, hypertension, and hypercholesterolemia [5]. Studies show that NO production declines with age not only in pathological states, but also in healthy aging [179,180]. Decreased activity or abundance of eNOS, reduced supply of Arg, increased concentrations of NOS inhibitors, and/or facilitated processes of NO scavenging or degradation could explain this decline. The endothelial NO production has a protective function against apoptosis by inhibiting the vital family of proteins caspases via the S-nitrosation of their critical cysteine [181,182]. Therefore, decreased NO generation in older individuals leads to reduced overall SNO protein signaling and age-dependent increased vulnerability to apoptosis.

The “free radical theory of aging” proposes that aging occurs due to the progressive buildup of harmful and irreversible DNA, protein, and lipid damage caused by ROS and RNS [183]. The levels of nitrated proteins, which serve as markers of nitrosative stress damage, tend to increase with age [184]. The specific 3-nitrotyrosine nitration of proteins involved in energy metabolism, calcium homeostasis, and signal transduction in aging skeletal muscle has been reported [183,185]. This specific modification deactivates such important enzymes as tyrosine hydroxylase, copper/zinc superoxide dismutase (Cu/ZnSOD), and manganese superoxide dismutase (MnSOD) [183]. Another study showed a notable escalation in protein oxidative and glycoxidative damage throughout the aging process of the human brain, reaching a critical point at the age of 60 [186]. S-nitrosation is a more specific oxidative modification of proteins. Unlike other oxidative proteins’ modifications, its level may depend not only on the activity of nitrosylases and denitrosylases, enzymes that specifically add or remove NO groups, but also on the whole homeostasis of S-nitrosated proteins in the cells [183,187].

**Figure 3:**
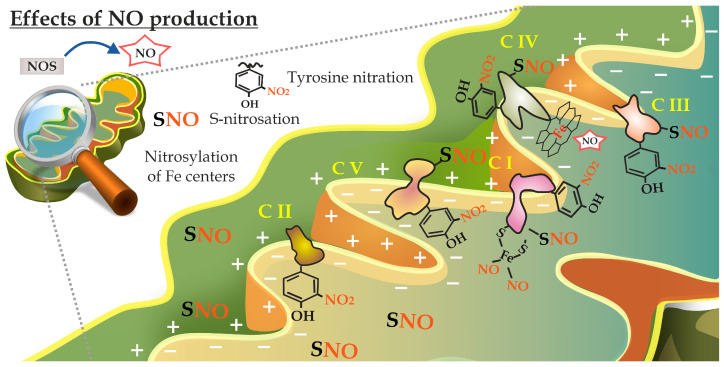
Reactions of NO with proteins in mitochondrial electron transfer chain (ETC): S-nitrosation, nitrosylation, and tyrosine nitration of respiratory chain proteins and other unspecified mitochondrial proteins, formation of dinitrosyl iron complexes of [Fe-S] clusters (C I), and nitrosylation of cytochrome c oxidase (C IV). Notation on the scheme: complex I (C I), complex II (C II), complex III (C III), complex IV (C IV), and complex V (C V).

Among other significant biological changes, aging is characterized by impaired energy metabolism [188]. In cells, mitochondria have several critical functions, including energy generation through oxidative phosphorylation, calcium homeostasis, the synthesis of lipid and iron–sulfur [Fe-S] clusters, the production of ROS, and the induction of apoptosis [189]. According to the “mitochondrial theory of aging,” the gradual decline in mitochondrial functions triggers a series of detrimental reactions that adversely affect all cellular organelles [183]. Mitochondria, being a source of superoxide anions within cells, provide a plausible foundation for the generation of peroxynitrite through its reaction with NO [181]. The decomposition of protonated peroxynitrite can cause an extensive amount of tyrosine nitration in mitochondrial proteins. This critical post-translational modification has been recognized in a variety of mitochondrial proteins, affecting their structure and function, and the metabolic destination of the proteins (Figure 3) [5,190]. NO has a strong affinity to Fe^2+^ and, even at remarkably low concentrations, binds to heme-containing cytochrome a3, forming a nitrosyl-heme center, which significantly inhibits mitochondrial respiration [190]. The direct reaction of NO with iron in the [Fe–S] centers in complexes I and II can damage them by removing iron, oxidizing the iron–sulfur bonds, or both [190]. Another way that NO can decrease the activity of complex IV is through noncompetitive binding to the dinuclear copper center found in cytochrome c oxidase [190]. The abnormal S-nitrosation of proteins in mitochondrial complexes could cause severe dysfunction in these organelles [182]. Dynamin-related protein 1 (Drp1) is one of the proteins that regulates mitochondrial fission. Its functionalization regulates several post-translational modifications like phosphorylation, ubiquitination, and S-nitrosation. The overproduction of NO triggers the S-nitrosation of another mitochondrial protein, parkin, leading to the upregulation of Drp1 [191]. Additionally, the influx of NO causes the phosphorylation of Drp1 Ser616 and an increase in mitochondrial fission [191]. These processes are linked to the development of Parkinson’s disease, a common neurodegenerative disease in elderly people [189]. The elevated level of S-nitrosated Drp1, often upregulated in aged cells, leads to mitochondrial fragmentation, impairs energy metabolism, and induces synaptic damage [189]. The S-nitrosation of the essential mitochondrial chaperone TRAP1 causes its subsequent degradation, changing mitochondrial homeostasis and causing metabolic reprogramming [192].

Another theory that attempts to explain the cause of aging focuses on the importance of cellular signaling responses in reaction to stress and damage. The dysregulation of signaling due to the formation of S-nitroproteins could serve as an example. S-nitrosoglutathione reductase (GSNOR) regulates intracellular levels of S-nitrosoglutathione and influences the extent of proteins’ S-nitrosation by denitrosylating target proteins [193,194]. Proteomic analysis revealed increased amounts of S-nitrosated proteins associated with neuronal and synaptic processes within the adult cortex and striatum [195]. These proteins play significant roles in the development of neurological and neurodegenerative disorders that often occur with age. For example, neutral cell adhesion molecule 1 (NCAM1), endocytic protein dynamin 1 (DNM1), soluble N-ethylmaleimide-sensitive-factor attachment protein receptors (SNARE proteins—SYB and SNAP25), and SNARE-associated proteins (CPLX1, SYN1, and MUNC18) can be mentioned. Additionally, the same study revealed a large number of S-nitrosated phosphatases and kinases [195]. These enzymes participate in a large number of signal transduction cascades. The S-nitrosation of these proteins induces an inhibitory effect on their activity, therefore modulating signaling in diverse cellular processes. Other modifications of the proteins, such as nitration and carbonylation, have been identified in the brains of aging rodents and humans [183,186,196].

Interestingly, GSNOR expression and activity are also affected by S-nitrosation since it is a cysteine-rich protein [194]. The decreased expression of GSNOR has been found in senescent cells during the aging process in both rodents and humans [197,198]. The elevated levels of S-nitrosated proteins involved in mitochondrial dynamics and mitophagy, such as parkin and Drp1, can be attributed to the decreased activity of GSNOR [197,198]. Unfortunately, conflicting reports regarding changes in GSNOR expression with age exist in the literature [199,200]. Zhang et al. reported the increased expression of GSNOR in the hippocampus of aging humans and mice [201]. Their results further showed the decreased S-nitrosation of hippocampal CaMKIIα protein, a key enzyme involved in memory formation and synaptic plasticity. Further studies suggested that GSNOR, through an S-nitrosation/denitrosation mechanism, may modulate other phenotype functions strongly influenced by age, such as muscle fatigue resistance and regenerative activity in the adult heart and liver. Y. Moon et al. revealed that GSNOR-deficient muscles were stronger and more fatigue-resistant, possibly due to the hypernitrosation of RyR1, a protein that regulates Ca^2+^ release and force development [202]. Other findings revealed that GSNOR-deleted cells exhibit a multifaceted cardioprotective response after post-myocardial infarction [203] and enhanced tissue regeneration after injury [204]. The up- or down-dysregulation of GSNOR is reported in a few other diseases such as cancer, cardiovascular, immune, asthma, and neurodegenerative diseases [194]. Due to such conflicting data, the GSNOR level may be subject to tight physiological regulation, making it a challenging therapeutic target [194,197].

The sirtuins (SIRT) family of proteins has been fully recognized as the main genes/protein machinery that regulates the mechanisms of aging and chronic diseases related to it, like Alzheimer’s disease, Parkinson’s diseases, and diabetes [90]. Sirtuins, functioning as NAD+-dependent histone deacetylases (HDACs), actively participate in various cellular processes including cell cycle regulation, fatty acid metabolism, gene transcription, and cellular stress response [90,205]. SIRT1 plays a crucial role in controlling cellular senescence and exhibits an anti-aging effect through the mitigation of inflammation and oxidative stress. The expression of SIRT is regulated by caloric restriction and physical activity.

Enhanced deacetylase activity in SIRT is linked to extended lifespan and improved healthspan in eukaryotes, whereas reduced activity is associated with an elevated susceptibility to aging-related diseases [91]. Despite the variety of positive age-regulated processes involved in SIRT, some conflicted data regarding their activity are present in the literature [90]. Enhanced NO production induces the S-nitrosation of SIRT1, which impairs the substrate binding and enzymatic activity of SIRT1. This post-translational modification is linked to the release of Zn^2+^ from the conserved zinc-tetrathionate site in sirtuins, leading to the disruption of the protein’s α-helical structure [206]. The S-nitrosation of SIRT1 can cause an abnormal activation of p53 and NF-kB that can contribute to various age-related disorders [207]. The denitrosation of SIRT1 can only occur in the presence of Zn^2+^ ions, and this process can fully restore the SIRT1 activity.

Aging is a cumulative process wherein damage accumulates over time to a critical point, leading to different age-related diseases and mortality. NO exerts regulatory effects on multiple cellular processes during aging. Several natural and synthetic therapeutic agents that affect NO signaling are tested as possible intervention strategies to decrease aging [5]. Their activity is often based on the increase in the bioavailability of NO through upregulating the expression of eNOS or nNOS, inhibiting the activity of iNOS, and suppressing the production of ROS and pro-inflammatory cytokines. However, considering the complicated involvement of NO in the aging process, a multitarget agent that can modulate NO levels, reduce chronic inflammation, decrease oxidative stress damage, and improve immune system functionality would have a beneficial effect on human health.

## 4. Sensing

The early detection and quantification of in vivo NO remain challenging due to NO’s instability, rapid reactivity, and low concentration in biological systems. Existing methods for detecting NO are often insufficient, demanding the development of innovative, non-invasive diagnostic tools capable of identifying disease progression at its early stages. This section focuses on the various techniques employed for detecting NO in these contexts, emphasizing their application, relevance, and potential for evaluating disease progression.

### 4.1. Cardiovascular System

Identifying the progression of cardiovascular disease early on remains a significant challenge in modern medicine, as current diagnostic methods typically detect the condition only in its advanced stages. Detecting the disease at these later stages results in less effective treatment outcomes. This fact underlines the need to develop and apply non-invasive techniques capable of recognizing the initial phases of cardiovascular illness. Existing diagnostic methods are not always reliable, and the precise etiology of the different diseases remains poorly understood [208]. Designing an accurate nitric oxide sensor for the human cardiovascular system is challenging due to several factors related to NO’s properties, sensor characteristics, and imaging limitations. Reported NO concentrations in tissues vary between studies and are influenced by the quantification methods used. Due to the broad concentration range (pM–µM), there is no universal sensor for NO that can be effectively used across different cells or tissues under various physiological and pathological conditions [209].

One approach of evaluating NO levels in cardiovascular diseases is through analyzing the patient’s exhaled breath. The ability to diagnose and treat various diseases using NO levels in exhaled breath has been significantly advanced by the development of accurate measurement technologies. NO was initially identified in exhaled air in humans, rabbits, and guinea pigs in 1991 using the chemiluminescence method [210]. Studies have investigated fractional exhaled NO as a potential biomarker for several diseases, including pulmonary hypertension [211,212], atherosclerosis (in patients with stable ischemic heart disease) [213], and adult congenital heart disease [214]. The chemiluminescence method is considered a “gold standard” technique when it comes to the detection of gas phase NO [215]. This technique involves the reaction of NO with ozone (O_3_) within a vacuum chamber, resulting in the formation of electronically excited nitrogen dioxide (NO_2_*). As NO_2_* molecules relax to their ground state, they emit photons that are detected by a photomultiplier tube, generating a signal that is linearly correlated with the NO concentration in the sample when O_3_ is in excess. Chemiluminescence equipment offers several key advantages, including a detection threshold of parts per billion, fast response times of 0.5 to 0.7 s, and the ability to analyze exhaled breath directly in situ. The chemiluminescence analyzers currently on the market are Sievers NOA 280i (GE Analytical Instruments, Boulder, CO, USA), Logan model LR2149 (Logan Research, Rochester, UK), NIOX (Circassia, Oxford, UK), and CLD 88 (Eco Medics, Duernten, Switzerland) [216]. Another method of analyzing exhaled breath is electrochemical detection, which functions by converting the concentration of a gas into an electrical signal. The breath sample is transferred to the sensor, where the target gas undergoes a chemical reaction facilitated by an active catalytic sensor, resulting in a measurable change within the electrical circuit. The signal is directly proportional to the partial pressure of NO, and consequently, to the NO concentration in the sample [215]. Achieving optimal NO selectivity and sensitivity in exhaled breath samples requires careful consideration of catalyst and electrolyte composition, along with a specialized chemical filtration system. Electrochemical methods often face difficulties in terms of selectivity, stability, and operational lifespan. Several commercially available electrochemical and infrared sensor devices include the NIOX VERO (Circassia), NObreath (Bedfont Scientific, Maidstone, UK), and Vivatmo-PRO (Bosch Healthcare Solutions GmbH, Waiblingen, Germany) [216].

**Figure 4:**
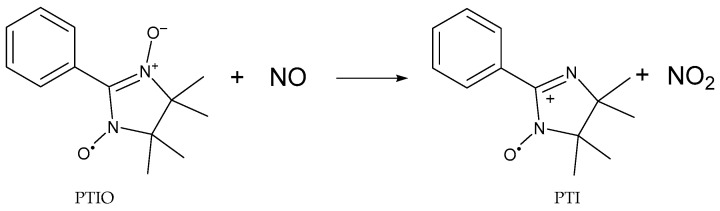
Reaction of PTIO with NO, leading to PTI (Pan et al. 2015 [217]).

Furthermore, extractive electrospray ionization mass spectrometry has been investigated as a potential method for analyzing breath samples [217]. The concentration of NO is determined by measuring the response of the product formed from the reaction between 2-phenyl-4,4,5,5-tetramethylimidazoline-1-oxyl-3-oxide (PTIO) and NO molecules using electrospray ionization mass spectrometry. In this simple chemical reaction, PTIO oxidizes NO, producing NO_2_ and 1-oxyl-2-phenyl-4,4,5,5-tetramethylimidazoline (PTI) (Figure 4). While this method exhibits high selectivity and a low limit of detection (0.02 ppb), and is less susceptible to matrix effects and ion source contamination, its practical application for online measurement is limited by the extended sample collection process and the necessity of using solvents.

Another method of examining nitric oxide in breath samples is based on laser technology [218]. Laser spectroscopy has emerged as a leading choice for high-performance sensing research and application, owing to its rapid response times, high sensitivity, and specificity. Laser spectroscopy sensors typically consist of a laser source that emits light, a gas cell containing the sample to be analyzed, and a detection system to measure the interaction between the light and the nitric oxide molecules. Laser-based techniques, including tunable diode laser absorption spectroscopy (TDLAS), the Faraday rotation spectroscopy (FRS), quantum cascade laser (QCL) technology, cavity-enhanced absorption spectroscopy (CEAS), and quartz-enhanced photoacoustic spectroscopy (QEPAS), offer high selectivity and quick response times for detecting target compounds, but the requirement for laser sources can lead to a substantial rise in instrument costs, along with challenges related to spectral degradation [219]. Recent applications of laser-based techniques offer the detection of nitric oxide isotopes and the simultaneous detection of different molecules such as carbon dioxide [220,221]. In summary, due to its simplicity, speed, non-invasive nature, and widespread availability, the procedure of analyzing NO in exhaled breath can serve as a valuable diagnostic tool for clinicians, as well as a means of monitoring disease progression and treatment response.

In both cultured cells and in vivo settings, several methods are commonly employed for the detection of nitric oxide. Among these, electrochemical sensors are particularly versatile, being suitable for both in vivo and in vitro applications. In an electrochemical sensor, nitric oxide is detected through either electroreduction or electrooxidation processes. Due to the presence of numerous potential interferents, such as endogenous nitrite, ascorbic acid, hydrogen peroxide, and glutathione, the selectivity of electrochemical sensors depends heavily on the use of permselective membranes [222]. These membranes function by allowing only low molecular weight gases to pass through, by employing electrostatic charge repulsion to distinguish between positive and negative ions, or by using hydrophobic polymers that significantly enhance the selectivity for NO. The effectiveness of NO detection is closely linked to the choice of electrode materials and surface modifications, with recent advancements highlighting the use of graphene nanostructures to enhance electrocatalytic activity [223]. One of the first examples of the direct application of electrochemical sensors for NO detection in cardiovascular condition occurred in 2010, when a NO sensor mounted in a catheter was inserted into the great cardiac vein, allowing real-time NO detection in the coronary circulation under the influence of acetylcholine. This study confirmed the impairment of NO in patients with nonischemic dilated cardiomyopathy and, therefore, demonstrated the potential clinical utility of NO sensors in assessing endothelial function for people with heart disease [224]. In another instance, an NO sensor demonstrated the ability to detect NO in a thin layer of human umbilical vein endothelial cells (HUVECs) subjected to shear stress, which is one of the factors in the development of atherosclerosis [225]. In 2020, Li et al. successfully designed a flexible, biodegradable NO probe. This wirelessly operated electrochemical sensor was able to effectively measure NO concentrations in cultured cells and organs. Furthermore, when injected into a rabbit, it enabled the real-time monitoring of NO levels for several days [226].

Similarly, electrochemical nanosensors have been developed for the simultaneous, real-time measurement of both NO and ONOO^−^ at the single-cell level. These nanosensors, with diameters ranging from 200 to 300 nm, have an exceptional detection limit of 10^−9^ M and a response time below one millisecond. Their ability to assess the NO/ONOO^−^ balance in normal versus dysfunctional endothelial cells revealed significant imbalances in hypertensive and diabetic rat models, where reduced NO and elevated ONOO^−^ levels indicated endothelial dysfunction. This application underscores the diagnostic potential of these nanosensors in detecting early signs of cardiovascular diseases and guiding targeted therapies [227]. In general, this development highlights the increasing versatility and effectiveness of electrochemical sensors and nanosensors in accurately monitoring NO and related molecules in a variety of biological environments, supporting both basic scientific research and practical clinical applications in cardiovascular health.

Another approach for detecting NO involves the use of molecular fluorescent probes. A number of organic and transition-metal complexes have been described in the literature concerning fluorescent NO detection [228]. The most well-known compounds for NO detection involve the utilization of o-diamino aromatics under aerobic conditions [229]. o-Diamino aromatics react with NO in the presence of oxygen to form fluorescent triazole derivatives (in the absence of NO, the fluorescence of the fluorophore is quenched by photoinduced electron transfer). When it comes to fluorescence-based NO detection in vivo, the imaging of NO is limited by an inability to specifically introduce the active sensor to the tissue of interest, with quantification typically being performed postmortem. A recent example of a fluorescent probe used in studies related to cardiovascular diseases could be a two-photon fluorescent molecular probe that is able to detect NO in live cells [230]. DANPY-NO is a 1,8-naphthalimide two-photon fluorophore conjugated to an o-phenylenediamine unit and a carbonyl-piperazine fragment. It is characterized by good photostability, pH insensitivity, and high quantum yield. The probe was effectively employed for the selective detection of intracellular endogenous NO generated by iNOS and eNOS across various cell types, including mouse macrophages, human leukemic cells, primary mouse macrophages, and endothelial cells. Chen et al. [231] developed a BODIPY-based (boron-dipyrromethene) fluorescent probe that enabled the study of the interplay between NO and glutathione. This probe exhibited yellow fluorescence in the presence of NO, followed by a red shift upon reaction with GSH, marking the first instance of a probe that could simultaneously detect these two molecules. The authors successfully visualized NO-induced GSH upregulation in pravastatin-treated HUVECs. In a related advancement, Liu et al. introduced an anthracene carboxamide-based sensor, AC-SA, which represents the N-nitrosation-based ratiometric sensor. Ratiometric probes offer an internal correction mechanism to minimize experimental artifacts resulting from factors like instrumentation and photobleaching. The AC-SA sensor was effectively utilized for endogenous NO fluorescence imaging in RAW 264.7 macrophage cells [232]. In addition to analyzing exhaled breath samples, mass spectrometry—specifically, membrane inlet mass spectrometry (MIMS)—enables the real-time, continuous monitoring of dissolved nitric oxide (NO) [233]. MIMS employs membranes that are selectively permeable to uncharged, low molecular weight molecules such as NO. Polymer membrane acts as a barrier between the aqueous sample and the vacuum chamber of the mass spectrometer. Volatile compounds dissolved in the sample diffuse through the membrane into the vacuum system, where they are analyzed by the mass spectrometer. A key advantage of MIMS lies in its stability, as it is minimally affected by temperature fluctuations, while also facilitating the simultaneous detection of other gases such as oxygen and carbon dioxide [234]. According to Tu et al., membrane inlet techniques can be effectively used for physiological measurements in both in vitro and in vivo settings, with the probe capable of being inserted into blood vessels [234].

### 4.2. Neurodegenerative Disease

**Figure 5:**
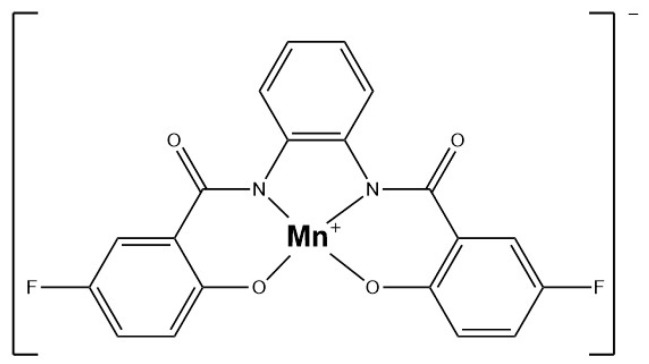
Structure of MRI probe—MnL1F (Barandov et al., 2020 [242]).

Nitric oxide, due to its versatile role in the nervous system as a biological mediator, modulator, and effector, holds significant potential as a biomarker for the progression and diagnosis of neurodegenerative diseases. Some of the previously used methods involve the detection of NO in cerebrospinal fluid (CSF) by electron paramagnetic resonance (EPR) [235,236,237] or by measuring NO oxidation products like nitrite [238,239,240]. However, recent developments focus on the in situ detection of NO. Among the methods for direct detection, electrochemical sensors are highly effective. One example is the metalloporphyrin nanosensors constructed by Alsiraey et al. [241], which are suitable for the single-cell detection of NO. The sensor is based on carbon fibers that provide a conductive base and is coated with a layer of metalloporphyrin to enhance selectivity and sensitivity to NO. The nanosensor works through an electrochemical reaction wherein NO, released by neurons or other cells, is oxidized at the sensor’s tip. This reaction generates an electrical current proportional to the concentration of NO. The current is measured using a three-electrode system consisting of a working electrode (the NO sensor), a reference electrode (Ag/AgCl), and a counter electrode (platinum). Numerous novel probes have been developed to facilitate the in situ imaging of NO and ONOO^−^ in both in vivo and in vitro models, with the goal of advancing diagnostic tools for neurodegenerative diseases. One example of NO in situ imaging, developed by Brandov et al., is a manganese-based probe MnL1F called NORA (NO-responsive agent) (Figure 5) [242]. This probe enables specific NO detection using MRI techniques due to NO-induced reduction in longitudinal relaxivity (r1) of the manganese (III) N,N′-(1,2-phenylene)bis(5-fluoro-2-hydroxybenzamide) complex. NORA was tested in vivo in a lipopolysaccharide (LPS)-induced neuroinflammation model using Sprague−Dawley rats. The probe was successfully applied as a contrast for NO detection in the inflamed brain tissue.

**Figure 6:**
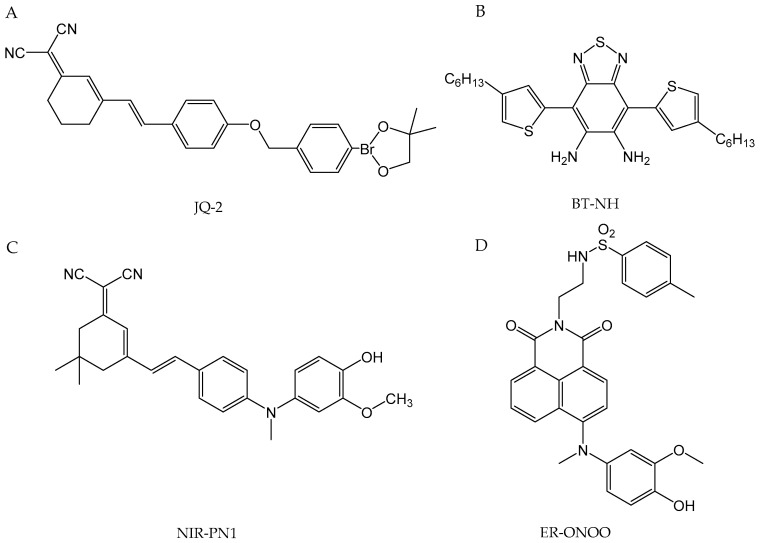
Structures of NIR fluorescent probes: (**A**)—Kang et al., 2022 [243]; (**B**)—Weng et al., 2019 [245]; (**C**)—Sun et al., 2020 [246]; and (**D**)—Yan et al., 2021 [244].

There is also great progress in the development of fluorescence-based probes. These probes are frequently characterized by the use of two-photon excitation and spectrum shifts into the NIR range, enabling deep tissue imaging—essential for in vivo studies [243,244,245,246], e.g., a NIR fluorescent probe JQ-2 (Figure 6A) [243] that is suitable for imaging ONOO^−^ in PD models. Probe structure is based on dicyanoisophorone-derivative fluorophore, which is quenched by a benzeneboronic acid pinacol ester moiety also responsible for a specific reaction with ONOO^−^. It was applied for a ratiometric measurement of ONOO^−^ in human cervical cancer (HeLa) cells and zebrafish rotenone-induced PD, offering both high sensitivity and selectivity with a low detection limit. Another example is the BT-NH probe (Figure 6B), which targets NO by standard o-phenylenediamine N-nitrosation and is characterized by deep-red fluorescence thanks to benzo-bis(1,2,5-thiadiazole) fluorophore, enhanced by the formation of triazole. BT-NH shows promise in the diagnosis of PD, as it was tested in human neuroblastoma cells (SH-SY5Y) and Parkin-Null Drosophila [245]. One more example is the NIR-PN1 probe (Figure 6C) that demonstrated an excellent sensing performance of ONOO^−^ and the ability to penetrate the blood–brain barrier (BBB) in PD in vivo models such as Drosophila, C. elegans, and the brains of C57BL/6 mice. NIR-PN1 utilizes dicyanoisophorone-based NIR fluorophore quenched through PET mechanism via p-aminophenol moiety that is released after a reaction with ONOO^−^. This highlights its significant potential not only to explain the biological roles of peroxynitrite in PD, but also for the early diagnosis and treatment of the disease [246].

**Figure 7:**
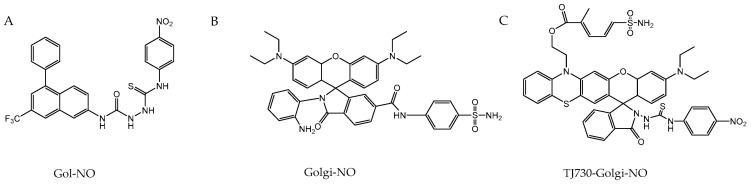
Structures of Golgi-targeted fluorescent probes Gol-NO: (**A**)—Zheng et al., 2023 [251], (**B**)—He et al., 2022 [250], and (**C**)—Xi et al., 2024 [252].

Many of these probes address the issue of low NO concentration by specifically targeting organelles such as lysosomes and mitochondria [247,248,249]. A proper example is the ER-ONOO probe (Figure 6D) with a two-photon excitation mechanism, targeting ONOO^−^ generated in the endoplasmic reticulum (ER) [244]. For targeting ER, Yan et al. used p-toluenesulfonamide linked to 1,8-naphthalimide fluorophore quenched by the ONOO^−^ sensing group—4-amino-2-methoxyphenol. It was studied in PD models of rat clonal pheochromocytoma cells (PC12 cells) and C. elegans. Recently, Golgi-targeted probes have emerged as powerful tools in diagnosing neurodegenerative diseases like Alzheimer’s and Parkinson’s [250,251,252]. Unlike traditional probes that typically monitor NO concentration changes in the cytoplasm or mitochondria, Golgi-targeted probes focus on detecting dysfunctions within the Golgi apparatus. Given that the Golgi apparatus is affected in neurodegenerative diseases and is rich in nitric oxide synthase [253,254], it is recognized as a critical site of early pathological changes in these conditions. Probes such as Gol-NO (Figure 7A) [251] and Golgi-NO (Figure 7B) [250] have been studied in vitro and in vivo in neurodegenerative disease models. Gol-NO is a turn on/off probe, activated by the reaction of NO with the thiosemicarbazide group that is also responsible for quenching aminoquinoline-derivative fluorophore. Released thiosemicarbazide after the reaction with NO ceases to quench the fluorescence of 4-phenyl-2-(trifluoromethyl)quinolin-7-amine. Leftover aminoquinoline is characterized by a green fluorescence (maximum 520 nm) and is responsible for targeting Golgi apparatus. The probe was tested in a rotenone-induced PD model of PC12 cells and zebrafish, showing rapid response to NO. In Golgi-NO probe, for the purpose of the specific targeting of the Golgi apparatus, 4-sulfamoylphenylamide was used and linked to the 6-carboxyl group of 6-carboxyrhodamine B moiety. O-diaminobenzene attached to the 2-carboxyl, after the reaction with NO, leads to the opening of the spirolactam, activating the probe’s green fluorescence (max 589 nm). Golgi-NO was tested in a cellular model of AD by the incubation of SH-SY5Y cells with amyloid β (Aβ42), indicating an increase in NO concentration in the Golgi apparatus. Although these probes demonstrate high specificity and sensitivity in NO detection, their primary limitation is a low Stokes shift, which does not align with the typical optical window required for imaging in deeper tissues. This limitation is addressed by the TJ730-Golgi-NO probe (Figure 7C), which features a large Stokes shift of 158 nm, making it more suitable for such applications [252]. Similar to Golgi-NO, TJ730-Golgi-NO utilizes a thiosemicarbazide for Golgi targeting and spirolactam ring opening in rhodamine TJ730, as well as oxadiazole formation after the reaction with NO, leading to the activation of the probe.

**Figure 8:**
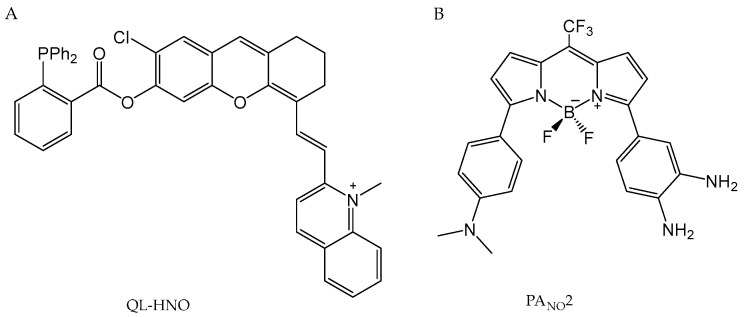
Structures of photoacoustic probes: (**A**)—Qi et al., 2024 [256] and (**B**)—Jiang et al., 2023 [257].

Recent developments in imaging technology have also introduced the use of photoacoustic imaging (PAI), a novel method combining optical excitation with ultrasonic detection [255]. The photoacoustic (PA) effect offers rich contrast, high resolution, and deep tissue penetration, making it an improvement over traditional fluorescence imaging. This advancement has gained significant interest in the detection and imaging of small biomolecules such as NO, ONOO^−^ and HNO [255]. Examples include a dual-modal probe Ql-HNO (Figure 8A) [256], which integrates both photoacoustic and fluorescence imaging due to a hemicyanine-based fluorophore turned on by the HNO-induced cleavage of triarylphosphine moiety. This probe allows for the tracking of HNO in vivo through complementary imaging techniques, offering high spatial resolution and sensitivity. The PA_NO_2 probe (Figure 8B), developed by Jiang et al. [257], marks a significant breakthrough in brain imaging due to its ability to cross the blood–brain barrier. The PA_NO_2 CF3-BODIPY core of the probe exhibits a highly selective ratiometric response to NO, enabling the high-resolution imaging and quantification of NO levels and minimizing the interference from the background signal throughout the entire brain in PD models of living mice. The probe’s ability to image NO at depths of up to 8 mm in the brain facilitates detailed visualization of NO distribution. The probe also has great physicochemical properties, such as water solubility and low molecular weight, making it a highly promising tool for visualizing NO dynamics in neurodegenerative diseases.

Rather than in situ imaging, change in the levels of these metabolites can be measured in biological samples, like CSF and blood serum with chromatography or immunoassay methods [258,259,260]. For instance, 3-NT, a marker of nitrative stress, was controlled in CSF to assess its role in diseases like AD. In a study by Ryberg et al. [259], 3-NT levels in CSF were measured by gas chromatography with mass spectrometer detection (GC-MS). However, this study revealed that most patients with AD did not exhibit elevated 3-NT levels in their CSF, indicating that free 3-NT in CSF may not be a reliable biomarker for this specific disease, which contrasts with the situation in PD, where significantly higher concentrations of 3-NT in both CSF and serum were present [258,261]. In the case of these studies, 3-NT levels were measured using a commercially available 3-NT ELISA kit. Although the differences between 3-NT in AD patients were not significant, studies of the proteome of S-nitrosylated proteins by mass spectrometry in AD have demonstrated a correlation between the S-nitrosation of proteins critical for synaptic function and amyloid formation and the progression of the disease [262,263]. One notable technique, described by Seneviratne et al. [262], is the SNOTRAP method, which enables the detection of all S-nitrosylated proteins by specific reaction with the triphenylphosphine thioester probe linked with biotin, offering a comprehensive view of the SNO-proteome in brain tissues. Although this method has not yet been applied to biological samples like blood serum, it suggests that profiling the SNO-proteome could serve as a diagnostic tool and potentially identify therapeutic targets for early intervention in AD.

### 4.3. Sensing in Cancer

Determining NO concentration is vital in cancer diagnosis because it is involved in various biological processes that influence cancer development and progression. Different methods exist for detecting NO, including UV-visible spectroscopy, EPR, chemiluminescence, fluorescence, imaging, and electrochemical techniques [264,265]. Among these, fluorescent probes, photoacoustic techniques, and electrochemical methods are the most widely used due to their sensitivity, selectivity, and ability to provide the real-time monitoring of NO levels in biological systems.

A survey of the literature focused on cancer diagnosis indicates that the most used approach for NO determination is the employment of fluorescent probes [266,267,268,269,270,271,272]. These probes allow for the accurate and sensitive detection of this biomarker in cancer cells. Fluorescence imaging offers numerous advantages, including high sensitivity, selectivity, and resolution. Most organic probes developed for detecting NO are based on o-phenylenediamine and dihydropyridine units. Fluorescent probes are highly used because they can be customized to exploit NO’s properties under physiological conditions, making them highly valuable for NO detection both in vitro and in vivo.

**Figure 9:**
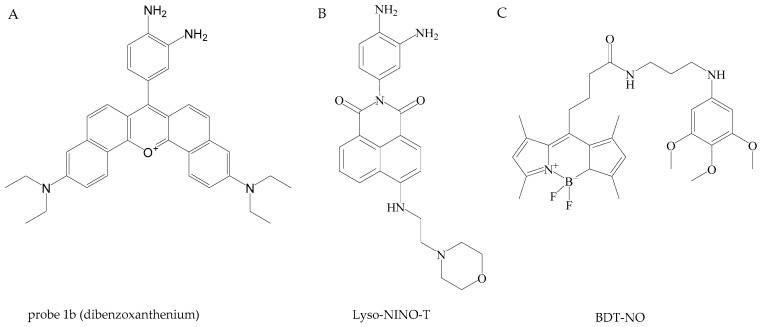
The structures of fluorescent probes and their chemical reactions: (**A**)—Liu et al., 2015 [267]; (**B**)—Yu et al., 2012 [272]; and (**C**)—Parisi et al., 2024 [269].

One notable example is a near-infrared (NIR) fluorescent probe containing two dibenzoxanthenium derivatives bearing a 3,4-diaminophenyl group developed by Liu et al. (Figure 9A) [228]. The enhanced fluorescence results from the suppression of PET between the diaminophenyl and benzoxanthene groups [265,267,273]. The probe’s effectiveness was demonstrated in HeLa cells. The experiment involved comparing the fluorescence intensity of cells incubated with the probe to those treated with the probe and subsequently exposed to 1-hydroxy-2-oxo-3-(3-aminopropyl)-3-methyl-1-triazene (NOC13)—an NO donor. The emergence of red fluorescence signified the conversion of the probe to a triazole derivative after reacting with NO. These findings underscore the probe’s efficiency in monitoring and imaging NO in living cells. Preliminary results indicate that such probes based on the dibenzoxanthenium scaffold hold considerable potential for further development in the cancer research. They could be instrumental in elucidating NO’s role in tumor biology and designing NO-targeted therapies.

Further research by Yu et al. (Figure 9B) introduced another important fluorescent probe, Lyso-NINO-T, that employs two-photon activation for imaging NO in the lysosomes of living cells. This probe is particularly useful for deep tissue imaging due to its modified naphthalimide fluorophore, allowing for reduced photodamage and enhanced targeting accuracy within lysosomes. The o-phenylenediamine component functions both as an electron donor, suppressing the probe’s fluorescence through PET, and as an NO trap, oxidizing the o-phenylenediamine fragment to the corresponding triazole derivative. The (aminoethyl)morpholine moiety specifically directs the probe to lysosomes within cells, ensuring that fluorescence activation occurs precisely where NO is located. The probe was utilized to monitor both exogenous and endogenous NO in living cells, specifically within the lysosomes of MCF-7 (breast cancer cell line) and RAW 264.7 (mouse macrophage cell line) cells. Its efficacy for detecting endogenous NO was tested in macrophages, where RAW 264.7 cells incubated with the probe for 12 h exhibited weak fluorescence, indicating the successful capture of endogenous NO in the macrophage lysosomes [272].

In addition, Parisi et al. developed a fluorescent probe BDT-NO (Figure 9C) meticulously engineered for the selective detection of NO in melanoma cell lines. It features a BODIPY fluorogenic unit that significantly increases in fluorescence upon reacting with NO, making it a powerful tool for live-cell imaging, particularly in melanoma cell lines. This design facilitates the efficient nitrosation of the active amine site by NO, aided by the intermediate formation of N_2_O_3_. Upon reacting with NO, the fluorescence quantum yield of the BODIPY unit markedly increases from 0.06 to 0.55. This significant enhancement in fluorescence is attributed to the suppression of PET, which would otherwise quench the fluorescence in the absence of NO. The probe can maintain a consistent fluorescence response across a broad pH range (4–11) and is highly reactive towards NO, although it also responds to ONOO^−^, another important oxidation product of NO. Additionally, it demonstrates minimal interference from other physiological substances such as glutathione. The probe’s efficacy was validated in B16 cells (melanoma cell lines), where it successfully detected NO in real time when used with a light-activated NO releaser. This experimental setup confirmed the probe’s rapid response and high sensitivity within a biological context, highlighting its potential for applications in live-cell imaging [269].

**Figure 10:**
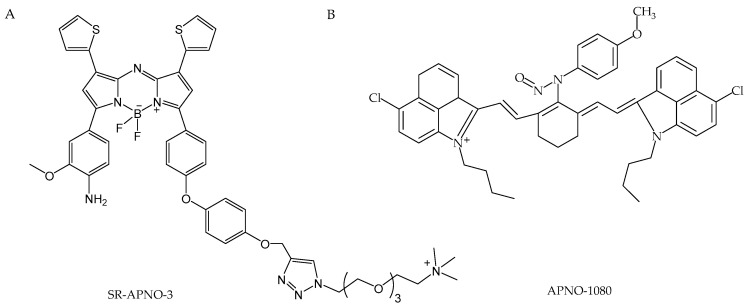
The structures of photoacoustic probes: (**A**)—Reinhardt et al., 2020 [274] and (**B**)—Lucero et al., 2021 [277].

In addition to fluorescent probes, photoacoustic probes are also a promising group of sensors tested in the context of NO detection in cancer diagnosis. These types of probes operate based on the photoacoustic effect, which converts light energy into sound waves. One notable example is a photoacoustic probe for NO detection, which utilizes the boron-azadipyrromethene dye platform (aza-BODIPY) through steric relaxation. The mechanism underlying the detection of NO in tissues hinges on enhancing the planarization of the aza-BODIPY dye, thereby improving its light absorption efficiency. The enhanced sensitivity and accuracy in NO detection are achieved by exploiting the differential light absorption properties of the probe before and after its reaction with NO, enabling the precise quantification of NO concentrations within tissues. Among several dyes based on the aza-BODIPY, a promising compound for photoacoustic NO detection in cancer is SR-APNO-3 (Figure 10A) [274]. The sensor exhibits a maximum light absorption wavelength of 790 nm before undergoing nitrosation (reaction with NO), whereas after nitrosation, this absorption peak shifts to 704 nm. This shift in wavelength is crucial for facilitating precise imaging using commercially available real-time photoacoustic tomographs. SR-APNO-3 is a much more sensitive successor of the APNO-5 sensor, which was successfully used to detect the concentration of NO relevant to the immune response [275]. Emerging evidence indicates that the tumor microenvironment subsequently reduces macrophage motility, trapping them within the tumor, which results in production of much lower levels of NO [276]. Therefore, the enhancement of the probe sensitivity for imaging cancer-derived NO is a key issue. The SR-APNO-3 probe, in comparison to parent APNO-5, exhibits a significant increase in ratiometric response to NO and was successfully applied to detected endogenous NO in a mouse model of breast cancer, where steady-state concentrations are significantly lower than those observed during immune responses [274]. This increased ratiometric response, achieved by comparing the intensities of two photoacoustic signals at different wavelengths, improves accuracy by accounting for variations in probe concentration and environmental factors. This leads to a more precise and sensitive detection of NO in complex biological settings [274]. Moreover, both SR-APNO-3 and its N-nitrosated derivative are also characterized by relatively good photostability, which increases its potential for use in NO bioimaging [274].

Another example from this group is the NIR-II APNO-1080 photoacoustic probe (Figure 10B), introduced by Lucero et al., that was designed for deep tissue imaging due to its absorption in the NIR-II range [277]. Besides deeper tissue penetration, the advantages of the application of the probes that exhibit absorption above 1000 nm are increased sensitivity and a better signal/noise ratio. The APNO-1080 probe is designed with an optimized p-anisidine trigger on the NIR-II cyanine platform, ensuring a significantly high extinction coefficient in absorption maximum at 1080 nm and no spectra overlap of N-nitrosation product with the probe. To evaluate the deep-tissue imaging capabilities of APNO-1080 in vivo, the researchers conducted PA imaging in an orthotopic breast cancer model (4T1-Luc) and a heterotopic lung cancer model (A549-Luc2). The lung cancer model, which mimics liver metastases, allowed for the imaging of cancer-derived NO in much deeper tissues.

The application of an electrochemical NO sensor is another available approach for detecting NO in cancer cells. For example, Abdelwahab et al. [278] developed a biosensor that integrates multiple enzymes—myeloperoxidase (MP), catalase (CAS), and SOD—on a nanocomposite layer of multi-walled carbon nanotubes-poly(5,2′:5′,2″-terthiophene-3′-carboxylic acid) (MWCNT-PTTCA), enabling the precise detection of NO released from cancer cells. Its mechanism relies on the electrocatalytic reduction of NO at the probe. Nitric oxide is generated through the interaction of NOS and L-arginine. In this system, MP serves as a catalyst for NO reduction, while CAS and SOD are utilized to prevent interference from H_2_O_2_ and O_2_^−.^ during the electrochemical reduction of NO. The practical application of the developed biosensor was evaluated by measuring the NO released from rat liver and cultured cells, specifically, human gastric cancer (AGS) and intestinal cancer (HT-29) cells. The validity of the probe was tested by the introduction of real samples (rat liver, AGS, and HT-29 cells) with L-Arginine to induce NO production. Experimental results demonstrated that adding real samples to a buffer solution (pH 4.0) without L-Arginine did not elicit a NO response. Conversely, the detection of NO in real samples was achieved in the presence of L-Arginine. The findings indicated that the biosensor can effectively detect NO released from this reaction and has the potential to be applied to biological samples [278].

A different innovative strategy for NO detection in single cells involves genetically encoded nitric oxide probes (geNOps), which provide advanced techniques for monitoring NO concentrations in cancer cells. These probes utilize the GAF (G cyclase, Adenylyl cyclase, FhlA) domain from a bacterial NO-binding protein fused with fluorescent proteins, allowing for the precise real-time tracking of NO dynamics at the subcellular level. When NO binds to the iron (II) center in the GAF domain, it leads to fluorescence quenching, providing a direct visual signal and enabling detailed insights into NO’s role in tumor processes and therapeutic responses [279]. A particularly noteworthy example is the NO-sensing gene switch system developed by Qin et al., designed for use in tumor-targeting bacteria. This biosensor holds significant potential for both diagnostic and therapeutic uses in cancer treatment. The system employs an attenuated strain of Salmonella enterica subsp. enterica serovar Typhimurium. The genetic circuit within this system is designed to detect NO via the NorR (nitrate and nitrite response regulator) protein, which then triggers the expression of the DNA recombinase Fimbrial phase variation recombinase E (FimE). This recombinase facilitates the unidirectional inversion of the promoter region (fimS), thereby activating the expression of specific target genes. Upon the colonization of tumors by S. Typhimurium, NO is produced by iNOS, which activates the gene switch system. This mechanism ensures that gene expression is precisely controlled within the tumor microenvironment and is specific to NO. Laboratory experiments showed that the presence of a chemical NO source, such as diethylenetriamine/nitric oxide (DETA/NO), effectively activates gene expression. Further in vivo studies validated the system’s efficacy in targeting tumor-specific NO generated by iNOS in a tumor model, highlighting its potential for clinical applications [280].

Overall, modern NO detection techniques play a key role in diagnosing and monitoring cancer and cardiovascular and neurodegenerative diseases. Advancements such as fluorescent probes, photoacoustic techniques, electrochemical biosensors, and genetically modified biosensors are improving our ability to visualize and quantify NO dynamics, enhancing our understanding of its role in disease progression. Future research focused on refining these techniques could lead to more effective and personalized therapeutic strategies.

## 5. Conclusions and Future Perspectives

More than forty years have passed since the discovery of NO as the elusive endothelium derived relaxing factor (EDRF), but various controversies still exist regarding its formation and the true identity of the signaling molecule, as well as its downstream effector sites and the mechanisms regulated by it. NO is classically derived from L-arginine-dependent NOS isoforms, but can also be formed endogenously via the serial reduction steps of nitrate and nitrite ions. Downstream signaling and functional effects link to both cGMP-dependent and cGMP-independent mechanisms, with protein thiol S-nitrosation as the most significant pathway. Knowledge about the nature and kinetics of the chemical interactions between NO or RNS and biological targets in a cellular environment is crucial not only to understand their role in biological signaling, but also to propose new drugs and therapeutic strategies for various diseases. Despite intensive interdisciplinary research towards understanding the NO biological activity and developing novel tools to control NO biosynthesis and metabolism in numerous disorders, particularly in the cardiovascular, nervous, and immunological systems, the number of approved clinical applications is limited. Innovative strategies that increase NO bioactivity and/or its specificity may have therapeutic potential for the treatment of diseases. One of the challenges with such groundbreaking drug candidates is to optimize their spatial and temporal delivery to achieve the desired effects without any unwanted disturbances in normal physiological redox signaling. The development of novel NO imaging agents will not only help with addressing such issues, but will also be a driving factor for establishing new diagnostic procedures and treatment protocols. Bioinorganic chemistry related to NO signaling and sensing can provide new ideas for the design of drugs and therapeutic strategies in the treatment of many civilization diseases, as well as help maintain the normal course of aging processes in the body.

## Figures and Tables

**Table 1:** Therapeutic strategies targeting NO signaling and dysfunction in various diseases.

Problem	Goal/Application	Target/Therapeutic Strategy	Examples of Compounds/Drugs
NO signaling disfunction in cardiovascular	Restoration of NO production and bioavailability	β3-adrenergic receptor agonists	Isoproterenol [27]
		NOX inhibition	GKT137831, ML171, and VAS2870 [28]
		Increased NOS activity	L-arginine, L-citrulline [29,30], arginase inhibitors [31], hydrogen sulfide [32], BH_4_ [33], folate [34]
		sGC stimulation	Vericiguat [36]
		PDE5 inhibition	Sildenafil [37]
		Inhalation of NO	Inhaled NO [38]
		nitrate/nitrite supplementation	organic nitrates and inorganic nitrites [39] beetroot juice [43]
Neuronal degeneration	Reducing RNS production	Selective inhibition of nNOS	nNOS inhibitors [46]
	Regulation of protein S-nitrosation in neurodegenerative diseases	S-nitrosation of GluN1 subunit of NMDAR	Nitromemantine [47]
	Prevent apoptosis	GAPDH nitrosation	CGP3466B [48]
Overproduction of NO in immune system	Block NO production	Targeting iNOS with specific and non-specific inhibitors	iNOS inhibitors [6,49]
COVID-19 respiratory complications	Improve oxygenation	Inhalation of NO	Inhaled NO [50,51]
Inflammation, autoimmune diseases, stroke	Reduce inflammation and modulate immune responses	Use of NO donors to achieve anti-inflammatory effects	GSNO, SNAP, SNP [52,53,54]
Atopic dermatitis	induced NO production	UV-induced NO production	NO produced by UV irradiation [55,56]
Regulation of NO level in cancer	Inhibit cancer cell proliferation	iNOS inhibition	L-nil [57] L-NMMA in combination with taxene [58] or pembrolizumab [59]
	Promoting cell death by ensuring high concentrations of NO	Upregulation of p53 pathway, mitochondrial cytochrome c release, ONOO^−^ generation	Organic nitrates Diazeniumdiolates Metal-NO complexes Furozans S-nitrosothiols Syndonimines [60]
		Inhibition of NF-κB pathway due to the S-nitrosation of p50	Diazeniumdiolate [61]
Cancer cell resistance to chemotherapy and radiotherapy	Enhance therapeutic efficacy of anticancer agents	Combine NO donors with chemotherapy or radiotherapy to sensitize cancer cells	NO donors combined with cisplatin, docetaxel, carboplatin [60,62]
